# Short-Term Probiotic Colonization Alters Molecular Dynamics of 3D Oral Biofilms

**DOI:** 10.3390/ijms26136403

**Published:** 2025-07-03

**Authors:** Nadeeka S. Udawatte, Chun Liu, Reuben Staples, Pingping Han, Purnima S. Kumar, Thiruma V. Arumugam, Sašo Ivanovski, Chaminda J. Seneviratne

**Affiliations:** 1 Center for Oral-facial Regeneration, Rehabilitation and Reconstruction (COR3), School of Dentistry, The University of Queensland, Brisbane, QLD 4006, Australia; uqnudawa@uq.edu.au (N.S.U.); chun.liu@uq.edu.au (C.L.); r.staples@uq.edu.au (R.S.); p.han@uq.edu.au (P.H.); s.ivanovski@uq.edu.au (S.I.); 2 Department of Periodontics and Oral Medicine, School of Dentistry, The Ohio State University, Columbus, OH 43210, USA; kpurnima@umich.edu; 3 La Trobe Institute for Molecular Science, School of Agriculture, Biomedicine and Environment, La Trobe University, Melbourne, QLD 3086, Australia; g.arumugam@latrobe.edu.au

**Keywords:** 3D salivary polymicrobial biofilm model, oral microbiome, streptococcus salivarius K12 (Ssk12), 3D melt electrowriting (MEW) medical-grade poly(ε-caprolactone) (mPCL), probiotic colonization

## Abstract

Three-dimensional (3D) scaffold systems have proven instrumental in advancing our understanding of polymicrobial biofilm dynamics and probiotic interactions within the oral environment. Among oral probiotics, *Streptococcus salivarius* K12 (*Ssk12*) has shown considerable promise in modulating microbial homeostasis; however, its long-term therapeutic benefits are contingent upon successful and sustained colonization of the oral mucosa. Despite its clinical relevance, the molecular mechanisms underlying the adhesion, persistence, and integration of Ssk12 into the native oral microbiome/biofilm remain inadequately characterized. In this pilot study, we explored the temporal colonization dynamics of *Ssk12* and its impact on the structure and functional profiles of salivary-derived biofilms cultivated on melt-electrowritten poly(ε-caprolactone) (MEW-mPCL) scaffolds, which emulate the native oral niche. Colonization was monitored via fluorescence in situ hybridization (smFISH), confocal microscopy, and RT-qPCR, while shifts in community composition and function were assessed using 16S rRNA sequencing and meta-transcriptomics. A single administration of *Ssk12* exhibited transient colonization lasting up to 7 days, with detectable presence diminishing by day 10. This was accompanied by short-term increases in *Lactobacillus* and *Bifidobacterium* populations. Functional analyses revealed increased transcriptional signatures linked to oxidative stress resistance and metabolic adaptation. These findings suggest that even short-term probiotic colonization induces significant functional changes, underscoring the need for strategies to enhance probiotic persistence.

## 1. Introduction

Microbial diversity is a key indicator of ecosystem stability and resilience, critical for host immunity, metabolic regulation, and the prevention of dysbiosis-associated conditions [1]. Probiotic therapy—also referred to as bacteriotherapy [2]—offers a biologically inspired approach to restore microbial equilibrium and enhance biodiversity through the administration of beneficial microorganisms [3,4,5,6,7]. In the context of oral health, probiotics hold considerable promise for the prevention and potential treatment of diseases by reversing microbial dysbiosis. Although a growing body of literature supports the clinical efficacy of probiotics in improving oral health parameters, the underlying mechanisms—whether driven by sustained colonization, direct modulation of resident microbiota, or transient activity—remain inadequately characterized. Long-term probiotic persistence and mucosal adhesion are considered pivotal to therapeutic success; however, the molecular and transcriptional frameworks that facilitate these processes in the oral cavity are still poorly understood. Notably, most randomized controlled trials (RCTs) investigating oral probiotic efficacy lack microbiological endpoints to definitively confirm colonization or its ecological impact [8]. While previous studies have reported changes in bacterial community composition [9,10], detailed insights into functional and ecological shifts remain limited.

Several recently characterized *Streptococcus* species have emerged as promising candidates for oral probiotic applications due to their efficient colonization capacity, favorable biocompatibility, role as pioneer colonizers, and amenability to precise experimental manipulation [11,12,13]. Among them, *Streptococcus salivarius* K12 (*S. salivarius* K12 or *Ssk12*), a natural commensal of the human oral cavity, has been extensively investigated for its therapeutic potential in oral healthcare [11]. A growing body of clinical evidence supports its efficacy in managing a range of conditions—including oral candidiasis, pharyngitis, tonsillitis, halitosis, otitis media, periodontitis, and dental caries—while preserving the ecological integrity of the native microbiota [11,14,15,16,17,18,19,20]. Recent studies have further elucidated the mechanistic basis of *Ssk12*’s probiotic function, highlighting its quorum sensing regulated proteolytic activity as a key factor in pathogen inhibition within the host microenvironment [21]. These insights have supported its commercial adoption as a widely used oral probiotic. *Ssk12* is one of the earliest microbial colonizers of epithelial surfaces in the oral cavity and nasopharynx, with a particular affinity for the dorsum of the tongue—a niche linked to the modulation of supragingival plaque composition and maturation [22,23,24,25]. In vitro selection strategies for this strain have employed hydroxyapatite adhesion assays as analogs for tooth surface colonization, alongside mixed-species biofilm models to simulate natural oral environments [26,27]. Collectively, these attributes position Ssk12 as a robust model organism for investigating microbial interactions, colonization dynamics, and functional alterations within the oral microbiome.

Melt electrowriting (MEW), an advanced additive manufacturing technique, enables the fabrication of microscale scaffolds with highly defined architectures that closely mimic the intricate anatomical features of biological tissues [28,29,30]. Our recent study has demonstrated that three-dimensional medical-grade polycaprolactone (3D MEW mPCL) scaffolds produced via MEW support the horizontal three-dimensional growth of saliva-derived biofilms, resulting in enhanced biofilm thickness, increased biomass accumulation, and improved polymicrobial retention [31]. Furthermore, we have shown that salivary biofilms cultured on 3D MEW mPCL scaffolds in vitro closely reproduce the microbial complexity and compositional fidelity of naturally formed oral biofilms [32]. These findings highlight the potential of MEW mPCL scaffolds as a robust model for studying dental biofilm behaviour under controlled experimental conditions. By more effectively replicating the complex environment of the salivary microbiome, these scaffolds may simulate key features of the early stages and structural characteristics of dental plaque development [33], thereby enabling detailed investigation of probiotic–microbiome interactions within the oral cavity. Based on this rationale, the objective of the present study was to examine the colonization dynamics of the probiotic *Streptococcus salivarius* K12 (*Ssk12*) on 3D MEW mPCL-supported salivary biofilms following a single administration and to assess its subsequent effects on the compositional and functional remodelling of the oral bacterial community.

## 2. Results

**Figure 1:**
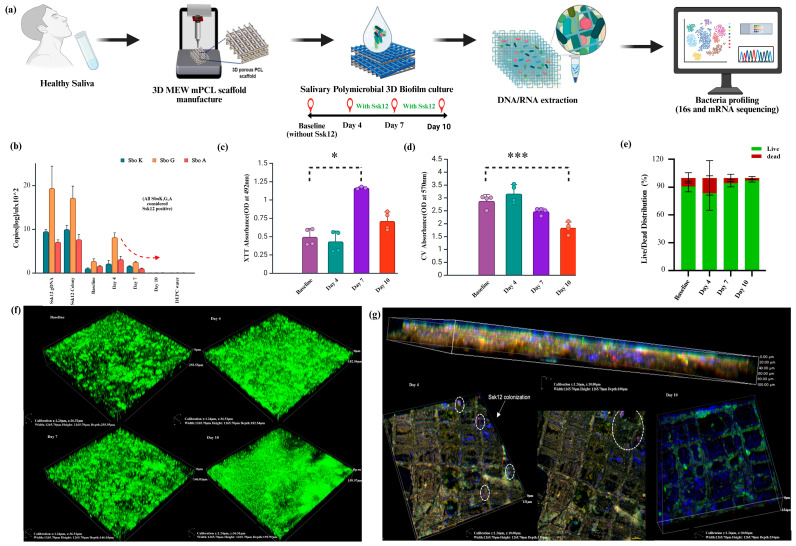
Characterization of salivary biofilms cultured on 3D MEW mPCL scaffolds with probiotic *S. salivarius* K12 (*Ssk12*) colonization. (**a**) Schematic illustration of the experimental workflow. Saliva from healthy human donors (n = 4) was used to generate multispecies biofilms on 3D MEW mPCL scaffolds. Biofilms were cultured with and without the probiotic *S. salivarius* K12 (*Ssk12*) and sampled at baseline (no probiotic), day 4, day 7, and day 10 for downstream DNA/RNA extraction and microbial profiling using 16S rRNA and meta-transcriptomic sequencing. (**b**) Quantification of *Ssk12* abundance across timepoints by targeting Ssk12 strain-specific genes *SboK*, *SboG*, and *SboA* via qPCR. The probiotic strain was detected at days 4 and 7, with a marked decline by day 10, suggesting transient colonization (*SboK*, *SboG*, and *SboA*, all positive, were considered *Ssk12*-positive). (**c**) XTT assay and (**d**) crystal violet (CV) assay showing biofilm metabolic activity and total biomass, respectively, on MEW mPCL scaffolds (pore size: 250 µm), as influenced by Ssk12 colonization. (**e**) Live/dead staining illustrating differences in biofilm thickness associated with Ssk12 colonization. (**f**) Representative 3D confocal laser scanning microscopy (CLSM) images of SYTO 9-stained salivary biofilms on MEW mPCL scaffolds at various timepoints, showing biofilm structural dynamics in response to *Ssk12* treatment. (**g**) High-resolution confocal z-stacks and side views showing spatial localization of adherent *Ssk12* using custom-designed smFISH probes (*Ssk12* indicated in pink) within the biofilm matrix. The fluorescent signal peaked on day 4 and declined by day 10, supporting transient colonization. * *p* < 0.05, *** *p* < 0.001. Abbreviations: MEW, melt electrowriting; mPCL: medical-grade poly(ε-caprolactone); XTT, tetrazolium salt assay; CV, crystal violet assay.

A schematic overview of the experimental design is presented in Figure 1a, with detailed protocols provided in Supplementary Section S1.

### 2.1. Transient Colonization of the Probiotic Bacteria into Oral Biofilm

We first assessed biofilm biomass and the presence of *Streptococcus salivarius* K12 (*Ssk12*) at baseline and following probiotic intervention on days 4, 7, and 10 using RT-qPCR, biofilm assays, and confocal imaging. Quantitative PCR revealed a significant increase in *Ssk12* levels by day 4 compared to baseline, followed by a gradual decline by day 10 (Figure 1b), with baseline samples exhibiting the lowest *Ssk12* expression, as expected (*SboK*, *SboG*, and *SboA*, all positive, were considered *Ssk12*-positive). The XTT assay (Tetrazolium salt, 2,3-bis (2-methoxy-4-nitro-5-sulfophenyl)-5-[(phenylamino) carbonyl]-2H-tetrazolium hydroxide) indicated higher metabolic activity in the 3D biofilm *Ssk12*-treated groups relative to baseline (Figure 1c), while the CV (crystal violet) assay showed a notable reduction in total biofilm biomass in the *Ssk12*-treated groups (Figure 1d). Viability assessments revealed higher percentages of live cells in the *Ssk12*-treated groups at day 7 and day 10 (Live: 94.16% ± 1.02%; Dead: 5.84% ± 0.02%) compared to baseline biofilms (Live: 91.61% ± 0.04%; Dead: 8.39% ± 0) (Figure 1e). Confocal imaging of live and dead bacteria after 10 days of biofilm culture showed that baseline groups without *Ssk12* treatment developed thicker live biofilms (day 4: 140 ± 5.8 μm; day 7: 180 ± 6.8 μm; day 10: 300 ± 10 μm) (Section S2, Figure S1) compared to *Ssk12*-treated groups (day 4: 170 ± 8.8 μm; day 7: 145 ± 6.8 μm; day 10: 155 ± 5 μm) (Figure 1f). Finally, single-molecule fluorescence in situ hybridization (smFISH) enabled visualization of adherent *Ssk12* cells on the MEW mPCL scaffold, showing that *Ssk12* distribution (indicated in pink) peaked on day 4 and declined by day 10 (Figure 1g).

### 2.2. Longitudinal Microbiota Composition Changes with SsK12 Probiotic Colonization

The impact of *Ssk12* colonization on oral biofilm composition was assessed using 16S rRNA gene sequencing. After quality filtering and chimera removal, a total of 1,207,393 high-quality reads were obtained, resolving 60 operational taxonomic units (OTUs) at the genus level across 16 biofilm samples. Rarefaction analysis confirmed that sequencing depth was sufficient for robust microbial diversity assessment.

**Figure 2:**
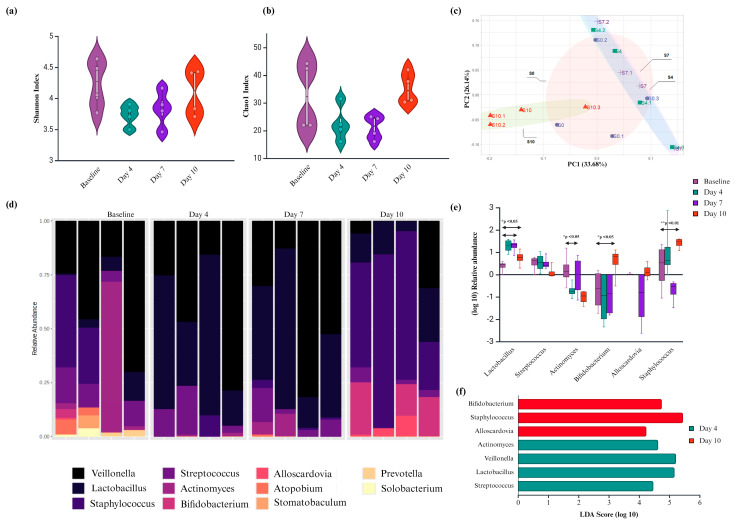
Compositional change of salivary biofilm following probiotic *S. salivarius* K12 (*Ssk12*) colonization on 3D MEW mPCL salivary biofilm. (**a**,**b**) Alpha diversity measures, including Shannon and Chao1 indices, were utilized to evaluate microbial diversity within each sample group. (**c**) Beta diversity analysis was performed to compare microbial community composition across the four groups: baseline (no *Ssk12*) and *Ssk12*-treated samples at days 4, 7, and 10. Principal coordinate analysis (PCoA) plots reveal distinct clustering patterns at each timepoint, indicating temporal shifts in microbial community structure. (**d**) Stacked bar plots depict the relative abundance of the top 11 bacterial genera across all groups. Each color represents a different genus, and the height of each segment indicates its relative proportion within the sample. (**e**) Log_10_-transformed relative abundance ratios are shown for the four groups. Bars represent the mean, and error bars denote standard deviation. Asterisks indicate statistically significant differences in abundance compared to the baseline group (*p* < 0.05). (**f**) Differentially abundant bacterial taxa identified using linear discriminant analysis (LDA) effect size (LEfSe) (LDA score > 2, q < 0.05), further validated by DESeq2 analysis. The LDA score reflects the magnitude of differential abundance, with higher scores indicating greater group-specific enrichment. * *p* < 0.05, ** *p* < 0.01.

Alpha diversity, as measured by Shannon and Chao1 indices, showed no significant within-sample differences across timepoints (Kruskal–Wallis, *p* > 0.05) (Figure 2a,b), although transient declines were observed on days 4 and 7, followed by recovery at day 10. In contrast, beta diversity analyses based on Bray–Curtis and Jaccard distances revealed significant shifts in microbial community structure over time (PERMANOVA, *p* < 0.05) (Figure 2c), with samples from day 10 clustering distinctly from earlier timepoints.

Sparse PLS-DA identified key genera driving these compositional changes, with *Alloscardovia*, *Bifidobacterium*, and *Staphylococcus* enriched at day 10, while *Streptococcus*, *Lactobacillus*, and *Rothia* were more abundant at earlier timepoints (Section S2, Figure S2). Overall, the phyla *Bacteroidetes*, *Firmicutes*, and *Actinobacteria* remained dominant across all samples (>84%), with *Veillonella*, *Lactobacillus*, and *Streptococcus* constituting the core genera (Figure 2d).

Notably, *Ssk12* colonization induced a significant early increase in *Lactobacillus* and a decrease in *Actinomyces* at day 4, followed by later enrichment of *Bifidobacterium* and *Alloscardovia* by day 10 (*p* < 0.05; LDA > 2; DESeq2) (see Section S2, Table S2). Although 16S profiling does not enable strain-level resolution, data suggest that *Ssk12* may have transiently displaced native *S. salivarius* strains, consistent with qPCR findings (see Section S2, Figure S1).

### 2.3. Functional Role of Ssk12 Colonization in Oral Biofilm Modulation with a Stronger Link to Metabolic Redox Regulation

**Figure 3:**
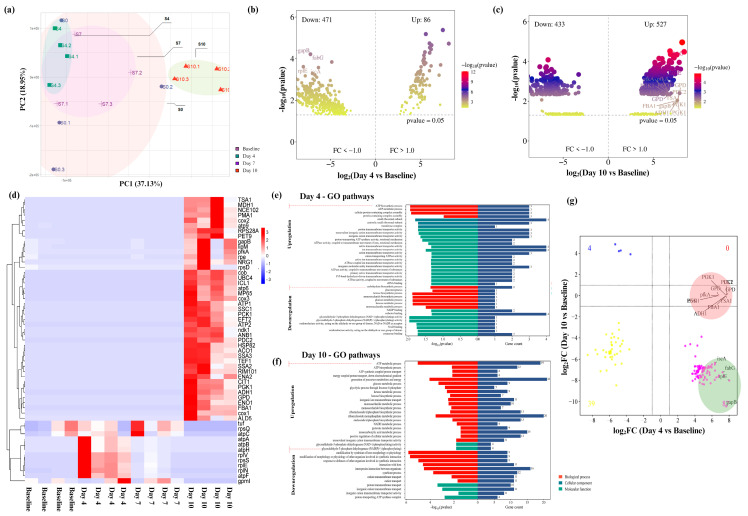
Functional changes of salivary biofilm following probiotic *S. salivarius* K12 (*Ssk12*) colonization on 3D MEW mPCL salivary biofilm. (**a**) Principal component analysis (PCA) of the gene expression profile (total genes = 7220). (**b**,**c**) Volcano plots displaying differentially expressed genes (DEGs; abs(log2FC) > 1, q < 0.01) on days 4 and 10, respectively. (**d**) Cluster heatmap showing the distribution of the top 55 DEGs in each sample. (**e**) Gene ontology (GO) pathways significantly enriched by upregulated and downregulated DEGs on days 4 and 10 (q < 0.05), categorized by molecular functions (MF), cellular components (CC), and biological processes (BP). (**f**) Scatter plot comparing log2FC values of shared DEGs between the day 4 group (highest *Ssk12* colonization) and the day 10 group (decline in *Ssk12* colonization). Red circled dots indicate positive log2FC values (Venn diagram in 3c), while green circled dots indicate negative log2FC values (Venn diagram in 3b), primarily associated with energy-demanding cellular path. (**g**) Generalized linear model (GLM) analysis demonstrated a strong inverse correlation in gene expression profiles between day 4 and day 10 (ρ = −0.85, *p* < 0.01); indicative of temporally opposing transcriptional responses. This pattern was marked by antiparallel regulation of key genes, wherein those upregulated on day 10 (positive log_2_FC; red circles) were significantly downregulated on day 4 (negative log_2_FC; green circles). Thirteen genes displayed statistically significant interaction effects across timepoints, many of which were linked to energetically demanding cellular functions.

To explore the functional impact of *Ssk12* colonization on the oral microbiome, we conducted a comprehensive transcriptomic analysis to identify differentially expressed genes (DEGs) at key timepoints. On average, 71,046 genes were detected per sample, with 7220 KEGG and GO categories annotated, indicating a robust functional landscape of microbial activity (see Section S2, Figure S3). Principal component analysis (PCA) revealed clear separation of the samples on days 4 and 10 (Bray–Curtis PERMANOVA, *p* = 0.001), while baseline samples exhibited greater individual variability (Figure 3a).

Comparative transcriptomic profiling revealed 557 DEGs at day 4 and 970 DEGs at day 10 relative to baseline, indicating dynamic shifts in microbial gene expression during and after peak *Ssk12* colonization (Figure 3b–d). GO analysis demonstrated significant enrichment in metabolic pathways associated with amino acid biosynthesis and oxidative phosphorylation at day 4, corresponding to a microbial functional reprogramming favoring energy production and protein synthesis (Figure 3e,f) (see Section S2, Figure S4). PPI network analysis delineated two primary gene modules linked to these metabolic processes, reinforcing the notion of enhanced cellular activity during peak colonization (see Section S2, Figures S5 and S6). Concomitantly, pathways related to carbohydrate metabolism, particularly gluconeogenesis, were downregulated at this stage. In contrast, by day 10, there was a notable resurgence of pathways governing glucose metabolism and immune regulation (Figure 3f), indicative of a metabolic recalibration following probiotic decline and suggesting a transient yet functionally significant impact of Ssk12 on the oral biofilm environment.

Building upon our earlier findings, we further validated the presence of key regulatory nodes by identifying common hub genes through PPI network analysis that were central to the transition between peak and declining Ssk12 colonization. Specifically, genes such as *nsaS*, *graS*, *yclK*, *fabG*, and *sigH* were identified (see Section S2, Figure S7), all of which are implicated in metabolic adaptation and stress response pathways, highlighting their pivotal roles in sustaining microbial resilience during probiotic colonization and its subsequent decline. In parallel, generalized linear model (GLM) analysis revealed a strong inverse correlation in gene expression profiles between days 4 and 10 (ρ = 0.85, *p* < 0.01; Figure 3g) (see Section S2, Figure S7), characterized by antiparallel regulation of key genes—those upregulated at day 10 (positive log_2_FC; red circles) were notably downregulated at day 4 (negative log_2_FC; green circles). Notably, thirteen genes exhibited significant interaction effects, predominantly associated with energy-intensive cellular processes, further emphasizing their critical contribution to regulating the dynamic success or failure of *Ssk12* colonization within the oral biofilm.

## 3. Discussion

This study presents the first integrated assessment of compositional and functional alterations in the oral microbiome following a single administration of the probiotic strain *Ssk12*, utilizing a three-dimensional melt-electrowritten medical-grade poly(ε-caprolactone) (3D MEW-mPCL) ex vivo biofilm platform. *Ssk12* successfully incorporated into the developing biofilm and remained detectable for up to seven days post-administration. Temporal analysis revealed distinct microbiome shifts between day 4—corresponding to peak *Ssk12* colonization—and day 10, when colonization levels had declined. These findings underscore the capacity of *Ssk12* to modulate the oral microbial ecosystem, suggesting that even transient probiotic colonization can drive both structural and functional reconfiguration of the resident microbiota.

Consistent with native oral microbial ecology, the 3D MEW-mPCL salivary biofilm model developed in this study recapitulated a community structure dominated by early and late colonizers typically derived from human saliva [34]. As saliva serves as a primary reservoir for seeding biofilm/plaque development, the presence of key genera such as *Veillonella* (38%) and *Prevotella* (4%) in our model closely mirrored their reported abundances in healthy saliva—17.23% and 6.48%, respectively [35,36]. Additionally, the model demonstrated enrichment of *Streptococcus* (7.9%) and *Actinomyces* (8.2%), taxa integral to both initial adhesion and later biofilm maturation [37,38]. Together, these genera accounted for 58.1% of the total microbial composition, underscoring the model’s capacity to replicate the ecological succession observed in natural oral biofilms. Core microbiota analysis at baseline also revealed that *Veillonella*, *Streptococcus*, *Lactobacillus*, and *Prevotella* together accounted for over 77% of the relative abundance, aligning with prior findings from our laboratory using the same 3D salivary biofilm platform [31,32]. These results highlight the model’s ability to recapitulate the complex taxonomic and successional architecture of natural oral biofilms, reinforcing its translational utility for investigating microbial dynamics and therapeutic interventions [33].

First, we assessed how probiotic *Ssk12* colonization modulates oral microbiome diversity. Notably, microbial diversity was lower during the first seven days of colonization compared to day 10, which coincided with the decline in *Ssk12* levels. The relative abundances of *Lactobacillus*, *Streptococcus*, and *Actinomyces* progressively increased from day 0 to day 7 during *Ssk12* colonization, while a sharp rise in *Bifidobacterium* was observed by day 10. Interestingly, a similar trend was reported in a previous animal study [39], which showed a 30% increase in *Actinomyces* and *Streptococcus* in the healthy *Ssk12*-treated group, along with modest increases in *Lactobacillus* and *Bifidobacterium*. Collectively, these findings underscore the regulatory influence of *Ssk12* on oral microbiota composition. Notably, certain strains of *Lactobacillus*, *Streptococcus*, and *Bifidobacterium*—widely recognized as key probiotic genera [40]—were significantly impacted by a single administration of probiotic *Ssk12*.

This study demonstrates that *Ssk12* can effectively modulate the oral microbiome; however, rather than restoring the microbial community to its baseline composition, *Ssk12* establishes a distinct ecological profile, corroborating previous findings [39]. Importantly, the impact of probiotic therapy should be considered beyond changes in individual microbial strains, emphasizing the broader shifts across the entire microbial ecosystem in both healthy and dysbiotic states. In this context, our findings provide compelling evidence that even a single administration of probiotics can induce significant alterations in oral microbial diversity.

Next, we investigated how functional metabolic pathways are influenced by probiotic *Ssk12* colonization. The functional effects of probiotics on oral biofilms are often species-specific and depend on their antimicrobial potential [41]. In the present study, most differentially expressed genes associated with higher *Ssk12* colonization were linked to energy-intensive metabolic processes, including ATP metabolism, amino acid metabolism, and ribosome assembly. KEGG pathway analysis revealed that the majority of hub genes were interconnected through pathways related to amino acid metabolism, oxidative phosphorylation, oxocarboxylic acid metabolism, and ABC transporters. Amino acid metabolism plays a critical role in the growth of early colonizers such as *Streptococcus* spp., which rely on coaggregation to stabilize the expression of genes involved in amino acid synthesis and membrane transporters [35,36]. Increased *Ssk12* colonization appears to stimulate cell proliferation and biofilm biomass accumulation, likely driving the upregulation of ribosomal protein mRNAs essential for ribosome production and cell division [37]. Similarly, studies on mixed-species biofilms challenged with *Staphylococcus aureus*, *Pseudomonas aeruginosa*, and *Salmonella* spp. [15] reported overexpression of pathways related to homeostatic functions, including transcription and translation, protein trafficking, and nucleoside and phosphate metabolism, in the presence of *Ssk12*. Collectively, these findings suggest that *Ssk12* colonization modulates critical metabolic and regulatory pathways to support microbial community stability and growth.

We further examined the distinct functional pathways that are specifically regulated during the peak colonization and subsequent decolonization of *SsK12* within the oral microbiome. A generalized linear model (GLM) regression analysis was employed to examine antiparallel gene correlations (log2FC values) between day 4 (peak colonization) and day 10 (decolonization). The analysis revealed that genes associated with energy-intensive cellular processes exhibited antiparallel expression patterns, with certain genes upregulated on day 10 and downregulated on day 4. These pathways included metabolic adaptations such as gluconeogenesis and hexose metabolism, as well as redox balance mechanisms involving altered NADP/NAD ratios, which play critical roles in colonization success.

During peak *SsK12* colonization on day 4, reduced gluconeogenesis and hexose metabolism, along with altered NADP/NAD levels, facilitate energy redirection towards the utilization of alternative carbon sources [38]. While many *streptococcal* species are regulated by catabolite repression, which suppresses the metabolism of non-preferred substrates in the presence of glucose, *SsK12* exhibits metabolic flexibility by efficiently utilizing raffinose and galactose [21]. This adaptive advantage enables its sustained growth in low-glucose environments, promoting the proliferation of beneficial genera such as *Lactobacillus*, *Actinomyces*, and *Streptococcus* while inhibiting glucose-dependent pathogenic bacteria through the production of organic acids. Furthermore, enhanced purine nucleoside triphosphate metabolism supports metabolic flexibility, allowing *SsK12* to switch between energy and carbon sources depending on availability.

By day 10, coinciding with the decline *SsK12*, there was a marked metabolic shift toward glucose-dependent processes, including upregulation of glycolysis, nucleotide biosynthesis, and enzyme activities related to fructose-6-phosphate metabolism. These changes favor fast-growing, glycolysis-reliant microbes that may outcompete *S. salivarius* through increased metabolic efficiency, DNA repair, and protein synthesis capacity [37]. Concurrently, increased oxidative stress responses during this phase may create a less favorable redox environment for probiotic persistence. Collectively, these findings highlight redox balance, energy efficiency, and metabolic competition as critical determinants of sustained colonization.

Our results reveal that *SsK12* colonization is associated with distinct transcriptomic reprogramming of the 3D oral biofilm, characterized by the upregulation of oxidative phosphorylation, amino acid metabolism, and ribosomal biosynthesis pathways. These energy-intensive processes, particularly oxidative phosphorylation, are indicative of enhanced microbial activity and ATP generation, which may support biosynthetic demands and confer competitive fitness within structured biofilms [37]. The enrichment of amino acid biosynthesis—especially of branched-chain and aromatic amino acids—has been previously linked to probiotic-mediated enhancement of microbial resilience and mucosal barrier support [42], suggesting a generally beneficial or neutral role in the context of probiotic colonization. Moreover, the colonization phase did not coincide with activation of classical virulence-associated pathways (e.g., LPS biosynthesis, secretion systems), further supporting a non-pathogenic, ecologically adaptive phenotype [43]. Importantly, oxidative phosphorylation has been previously associated with redox homeostasis in oral biofilms, which may help suppress acidogenic and anaerobic pathogen overgrowth—key contributors to periodontitis [44]. Although these metabolic shifts suggest a favorable adaptation to probiotic presence, confirmation of downstream host benefits—such as reduced inflammation—requires further validation. Given the in vitro nature of this model, which lacks host immune and signaling components, future in vivo studies are essential to elucidate how these transcriptomic profiles influence host–microbe interactions and periodontal health outcomes.

From a translational perspective, our findings highlight the need for therapeutic strategies that enhance probiotic colonization and stability within the oral environment, with particular emphasis on modulating metabolic pathways to support sustained microbial persistence. Consistent with the findings of Horz et al. [24], our study demonstrated that *SsK12* exhibits a transient colonization profile, with a notable decline in abundance observed after 7 days. Horz et al. reported that *Ssk12* may persist on mucosal surfaces for up to three weeks, with a marked decrease after day 8, suggesting that repeated administration may be required to maintain effective colonization. Our results reinforce this notion, emphasizing the temporal limitations of single-dose probiotic delivery and underscoring the importance of sustained or repeated uptake strategies to ensure prolonged functional engagement within oral biofilms. Importantly, these insights also point to the potential of targeted prebiotic supplementation—such as polyphenols or amino acid-derived substrates—to support the metabolic demands of probiotic strains. By selectively enhancing energy-generating and biosynthetic pathways, such prebiotics could facilitate niche-specific persistence, strengthen host–microbe interactions, and extend the functional longevity of probiotic interventions.

This study further advances our understanding of probiotic dynamics in oral biofilms by elucidating the molecular and functional determinants of *Ssk12* persistence, including its impact on microbial composition and the enrichment of pathways related to oxidative stress and metabolism. Recent work by Cheng et al. [45] demonstrated that microbial metabolites and oxidative stress modulators can influence NF-κB activation, thereby impacting epithelial cell proliferation, apoptosis, and immune responses. While our current model lacks host epithelial components, the observed microbial functional shifts provide a compelling basis to hypothesize that probiotic-driven metabolic reprogramming may, in a host-integrated system, influence such pathways. Such findings highlight the importance of biomimetic in vitro systems—such as epithelial co-cultures or scaffold surface modifications—that more accurately replicate host–microbe interactions and support long-term probiotic integration. Beyond mechanistic insights, the 3D MEW mPCL scaffold model employed here also holds translational promise for diagnostic innovation. By enabling controlled simulation of probiotic colonization and community shifts, it serves as a testbed for evaluating nucleic acid-based detection platforms—such as the dual RPA–LFIA system described by Zhang et al. [46] for rapid, multiplex monitoring of microbial markers. These technologies could be leveraged for real-time surveillance of probiotic persistence, microbial dysbiosis, and therapeutic responses in oral health settings. Taken together, our model supports both therapeutic optimization and diagnostic development, laying the groundwork for microbiome-based strategies to improve oral and systemic health.

This study presents several limitations that warrant consideration. First, the pilot investigation was conducted using a relatively small sample size; however, the use of non-pooled saliva samples enabled the development of personalized biofilm models that captured inter-individual variability. While the limited cohort size may constrain generalizability, this approach was appropriate for the exploratory nature of the study, particularly given the expected biological homogeneity among healthy individuals. To mitigate the impact of the small sample size, we employed rigorous quality control measures—including technical replicates, high sequencing depth (>10 million reads/sample), and robust statistical analyses—consistent with best practices in metagenomic research. Future studies involving larger and more demographically diverse cohorts are necessary to strengthen statistical power and extend the applicability of these findings. Second, although the in vitro 3D biofilm model employed here offers a physiologically relevant platform, it does not fully recapitulate the multifactorial complexity of the in vivo oral niche. The absence of host-derived factors—such as immune mediators, salivary flow dynamics, epithelial cell interactions, and inflammatory signaling—limits the capacity to evaluate host–microbe interactions essential for niche sensing and microbial persistence. Probiotic strains like *S. salivarius* K12 are known to rely on host-specific cues for stable colonization, and their behavior in vitro may not fully reflect in vivo dynamics. Moreover, experimental parameters such as static versus dynamic culture, nutrient composition, oxygen gradients, and scaffold architecture can significantly influence biofilm development and microbial community behavior. While the melt electrowritten (MEW) polycaprolactone (mPCL) scaffolds used in this study offered structural consistency and a defined framework for biofilm growth, their synthetic and biologically inert nature may limit microbial adhesion and affect transcriptional responses. Nevertheless, the melt electrowritten (MEW) mPCL scaffolds used in this study offer distinct advantages by providing a defined three-dimensional architecture with micro-scale porosity, which serves as a physical ecological niche that facilitates initial bacterial attachment and biofilm development. As previously reported by our group [31], cross-sectional scanning electron microscopy (SEM) of saliva-cultured MEW250 scaffolds at day 10 revealed hallmark features of mature biofilms, including porous architecture, interconnected channels, and prominent exopolymeric bridges—indicating sustained microbial aggregation and matrix formation. These findings suggest that while *S. salivarius* K12 showed transient colonization, being absent by day 10, the biofilm continued to mature, likely dominated by other resident species. Although MEW mPCL scaffolds improve the structural support for biofilm formation compared to conventional PCL, they still lack key biochemical cues such as extracellular matrix proteins and host cell ligands. Finally, the study did not investigate the long-term functional outcomes following the observed decline in *SsK12* colonization. Further, a single-dose administration allowed for the controlled assessment of early colonization dynamics; future investigations should explore repeated or sustained delivery strategies over extended timeframes to better simulate clinical use and evaluate the durability of probiotic effects. Collectively, these limitations highlight the need for integrative, host-informed biofilm models and in vivo validation to comprehensively assess the therapeutic potential of oral probiotics.

## 4. Materials and Methods

### 4.1. Salivary Sample Collection and Establishment of Ex Vivo Community

Ex vivo biofilm cultures were established using saliva from four periodontally healthy individuals (Section S2, Table S1), yielding a total of 16 biofilm samples. These were cultivated on three-dimensional melt-electrowritten medical-grade polycaprolactone (3D MEW mPCL) scaffolds and monitored across four time points: baseline, and days 4, 7, and 10.

Participant recruitment and study procedures were approved by the Human Research Ethics Committee of The University of Queensland (HREC No. 2023000467/06/2024). Eligibility was determined based on the 2017 World Workshop on the Classification of Periodontal and Peri-Implant Diseases and Conditions. All participants exhibited no history of periodontal disease, probing pocket depths (PPD) ≤ 3 mm, and bleeding on probing (BOP) ≤ 10%. The study cohort comprised four individuals (two males and two females, 50% each), aged between 37 and 40 years (mean age: 38.5 ± 1.29 years). Each participant self-identified as Asian and demonstrated periodontal health, with PPD < 3 mm, mean BOP of 1.8% ± 0.54% (range: 1–2.2%), and a plaque index (PI) of 0.075% ± 0.07% (range: 0–0.18%) (see Section S2, Table S1).

Exclusion criteria included current tobacco use or cessation within the past 12 months, the presence of oral or systemic diseases, recent orthodontic treatment (within 12 months), recent antibiotic use (within 3 months), and the use of prebiotics, probiotics, or antiseptic mouthrinses within 3 months of sample collection. Participants were instructed to abstain from food and beverages for at least one hour prior to saliva collection. Unstimulated whole saliva samples were collected and preserved in 20% glycerol (final concentration), then stored at −80 °C until use.

### 4.2. Manufacture of Melt Electrowriting MEW PCL Scaffolds

Melt electrowriting (MEW) was used to fabricate 3D fibrous scaffolds from medical-grade PCL (Corbion Inc., Australia, PURASORB PC 08, Item# 1850006). Briefly, a syringe was placed into the MEW device to fabricate precise scaffolds with carefully regulated conditions, as previously optimized [31,32] (see Supplementary Section S1.1).

### 4.3. Salivary Biofilm Culture and Ssk12 Inoculation

Four healthy participants’ saliva was utilized to establish an ex vivo biofilm culture inoculum, which was subsequently cultured on 3D MEW mPCL surfaces. The ex vivo community was characterized using both 16S rRNA gene and mRNA sequencing techniques were applied to examine the modulation in bacterial DNA composition and active gene expression associated with varying levels of probiotic Ssk12 colonization.

Following our previously optimized protocols [31,32], mPCL scaffolds were sterilized using 70% ethanol for 15 min, followed by UV sterilization for 20 min, before being incubated with saliva as an inoculum for biofilm culture and subsequent *SsK12* inoculation. *Streptococcus salivarius* subsp. *salivarius* strain K-12 [DSM 13084] obtained from ATCC (University Boulevard in Manassas, Virginia, USA), was cultured using BHI broth (BD Biosciences) according to the manufacturer’s instructions provided (ATCC) (see Supplementary Section S1.4). The *Ssk12* mixture inoculum was prepared by combining saliva, 85% Brain Heart Infusion (BHI) broth, and *Ssk12* (100ul) to achieve a final dilution of 1:20. The ex vivo salivary biofilm culture was established using a mixture of defibrinated sheep’s blood (ThermoFisher Scientific, Waltham, MA, USA) and heart infusion (HI) media in a 1:1:8 ratio.

A total of 300 µL of the prepared mixtures were seeded into 48-well plates containing mPCL scaffolds and incubated for 10 days in an anaerobic chamber (A20 Whitley Anaerobic Chamber, Bingley, West Yorkshire, UK) at 37 °C under continuous agitation at 80 rpm. Biofilm culture media (~15 mL) were harvested from the MEW mPCL scaffold-grown biofilms on days 4, 7, and 10 for subsequent downstream analyses. The dosing level of strain K12 was chosen to achieve colonization levels comparable to those naturally occurring in the oral cavity for this bacteriocin-producing strain [24].

Biofilms were cultured both with and without *SsK12* inoculation on day 4, while only *S. salivarius* K12-inoculated biofilms were maintained for 10 days. Culture media were replenished on days 4 and 7, following a previously established optimization protocol [31,32]. The ex vivo biofilm formed on day 4 without *Ssk12* inoculation served as the baseline reference for comparative analysis. All experiments were conducted in triplicate per sample and repeated six times to ensure reproducibility and robustness of results.

### 4.4. Characterization of Salivary Biofilm Biomass

Biofilm biomass was evaluated using crystal violet (CV) and XTT assays, with live/dead staining employed to characterize biofilm biomass on MEW mPCL scaffolds via confocal microscopy, as previously described [47,48]. The methodology is described in detail in Supplementary Section S1.5.

### 4.5. Validation of Colonization Ssk12 in Salivary Biofilm Biomass Using Real-Time Quantitative PCR (RT-PCR) and smFISH (Single Molecule FISH)

A quantitative assessment was conducted to identify and measure the presence of probiotic *Ssk12* colonization within mature salivary 3D mPCL biofilms. This was achieved through the application of an RT-qPCR TaqMan-based assay designed to target three specific regions of the K12 sbo locus (*SboK*, *SboG*, and *SboA*, all positive, were considered *Ssk12*-positive), as previously described [49] (see Supplementary Section S1.6). *SsK12* gene expression normalized to the 16S housekeeping gene. Relative gene expression levels were determined via the 2^−ΔCt^ method.

Single-molecule fluorescence in situ hybridization (smFISH) combined with confocal microscopy was employed to determine the spatial localization of *Ssk12* within salivary biofilms grown on 3D MEW mPCL scaffolds. Custom-designed HuluFISH probes (Pixelbio, Heidelberg, Germany) were used to visualize *Ssk12* cells adherent to the 3D scaffold surface. Strain-specific probes targeting *Streptococcus salivarius* were labeled in red, green, and orange, while a customized probe specific to the K12 strain was labeled in pink. Hybridization and staining procedures followed the manufacturer’s protocol [50] (see Supplementary Section S1.6). Imaging was performed using a Leica SP2 confocal microscope (Leica Microsystems, Buffalo Grove, IL, USA) equipped with Z-stack acquisition capabilities. Three-dimensional reconstructions and image analyses were conducted using Volocity 5.0 software (PerkinElmer, Waltham, MA, USA) and ImageJ (version 1.54m) (National Institutes of Health).

### 4.6. Microbial Profile Using 16srRNA Sequencing mRNA Sequencing

Genomic DNA (gDNA) and total RNA were harvested from the biofilms at different time points, specifically at baseline (without Ssk12 inoculation), and days 4, 7, and 10 with *Ssk12* inoculation. The extraction was carried out using the PureLink™ Microbiome DNA Purification Kit in accordance with the manufacturer’s instructions (Invitrogen, Waltham, MA, USA). The RNA extraction was performed separately using an R RNAzol^®^ assay kit. In 16S rRNA sequencing, library preparation was conducted by amplifying the V3–V4 region. More details are provided in see Supplementary Section S1.7.

Pre-processing of raw 16S sequencing data was conducted following an established protocol, as previously described [32], with the detailed methodology provided in Supplementary Section S1. A total of 62 operational taxonomic units (OTUs) were identified from 16S rRNA-targeted amplicon sequences, clustered using an open-reference OTU picking strategy at a 97% sequence similarity threshold. Rarefaction curves demonstrated sufficient sequencing depth and saturation, supporting the reliability of downstream analyses. Taxonomic classification of representative sequences was performed using the Ribosomal Database Project (RDP) Classifier with a Bayesian algorithm to determine microbial community composition across taxonomic levels. Alpha diversity metrics—including Chao1, Shannon, and ACE indices—were calculated and visualized in R (version 4.2) using packages such as *phyloseq* [51], *ggplot2*, and Xia Lab. MicrobiomeAnalystR. Available at: https://github.com/xia-lab/MicrobiomeAnalystR (accessed on 3 July 2024). Beta diversity was assessed through Principal Coordinate Analysis (PCoA) based on Bray–Curtis dissimilarity and weighted UniFrac distances. Statistical significance in community structure differences was evaluated using PERMANOVA, while diversity indices were compared using non-parametric Wilcoxon and Kruskal–Wallis tests.

To examine genus-level relative abundance, OTUs detected in fewer than two samples or with less than 1% abundance in any sample were filtered out. Genera exhibiting significant changes in abundance were identified by converting relative abundances to absolute read counts using *MicrobiomeAnalystR* algorithms [52]. Taxa driving compositional differences across timepoints were visualized using sparse Partial Least Squares Discriminant Analysis (sPLS-DA) [53], with variable importance in projection (VIP) scores used to evaluate each genus’s contribution to group separation between timepoints with and without probiotic intervention. Weighted UniFrac distances were employed to incorporate both phylogenetic relationships and relative abundances. Differential abundance analysis among *Ssk12*-treated groups was performed using *DESeq2* [54], with *q*-values adjusted using the Benjamini–Hochberg procedure. Microbial biomarkers showing statistically and biologically meaningful changes were identified using the Linear Discriminant Analysis Effect Size (*LEfSe*) method [55], applying a log LDA score threshold of 2 and a significance cutoff of *p* < 0.05.

In mRNA sequencing, total RNA samples underwent ribosomal RNA (rRNA) depletion to enrich for messenger RNA (mRNA) transcripts, targeting both prokaryotic and eukaryotic rRNAs using the Ribo-Zero™ Plus kit (Illumina, San Diego, CA, USA, 20040529). The enriched mRNA was then fragmented and precipitated following the protocol described by [56]. RNA-Seq libraries were prepared using the Illumina Stranded Total RNA Prep Ligation kit with IDT for Illumina RNA UD Indexes (Illumina, 20040554), according to the manufacturer’s instructions (Document #1000000124514 v03, June 2022). The sequencing was conducted on an Illumina platform, and raw FASTQ files were generated using *bcl2fastq2* (v2.20.0.422). Adapter sequences were removed using default *bcl2fastq* parameters, and stringent quality control procedures were applied to the raw reads. Specifically, reads containing adapter contamination, more than 10% undetermined bases (N), or over 50% low-quality bases (Q ≤ 5) were excluded. Quality filtering and adapter trimming were performed using *fastp* and *Trim Galore* [57], ensuring high-confidence reads for downstream analysis. Following preprocessing, high-quality reads were assembled using *MEGAHIT* [58], an ultra-fast assembler optimized for metagenomic datasets. For functional annotation, assembled contigs were queried against the EggNOG and Pfam databases to assign functional gene categories. Gene abundances were quantified based on the cumulative abundance of contigs annotated to each functional group, providing a comprehensive overview of gene expression profiles across samples.

### 4.7. Identification of Differentially Abundant Genes and GO Functional Pathway Enrichment Analyses

Principal Component Analysis (PCA) was conducted using the *stats* package in R to evaluate the overall spatial distribution of gene expression profiles. Differentially expressed genes (DEGs) were identified based on an adjusted *p*-value < 0.05 and an absolute log fold-change (|log_2_FC|) ≥ 1.0, with upregulated genes defined as log_2_FC > 0 and downregulated genes as log_2_FC < 0. Statistically significant DEGs were visualized using a volcano plot generated with the *ggplot2* package (R version 4.2). The top 55 most significantly upregulated and downregulated genes were further illustrated in a heatmap constructed using the *heatmap* package.

Gene Ontology (GO) enrichment analysis for differentially expressed genes (DEGs) was performed using the DAVID database (Version 6.8) [59], categorizing terms into biological process (BP), cellular component (CC), and molecular function (MF), with statistical significance defined as false discovery rate (FDR) < 0.05. The STRING database [60] was employed to construct a protein–protein interaction (PPI) network for DEGs using a combined score threshold > 0.4. The PPI network was visualized using *Cytoscape* software (Version 3.7.2) [61], and significant modules were identified using the Molecular Complex Detection (MCODE) plugin with scores > 10. High-scoring modules were highlighted as critical components of the network, with significance defined by *p*-value < 0.05 and FDR < 0.05 (or <0.25 for extended analyses) (see Section S2, Figure S5).

### 4.8. Identification of Top Hub Genes and Antiparallel Gene Expression Between Peak and Decline of Probiotic Ssk12 Colonization

Hub genes were identified from the most statistically significant module network, described above, using the *Cytoscape* CytoHubba plugin (Version 0.1). Genes with a degree ≥ 10 were designated as hub genes, with statistical significance defined as an adjusted *p*-value < 0.05. The top six hub genes were those exhibiting the highest degree values. The Kyoto Encyclopedia of Genes and Genomes (KEGG) database, which links genes to functional pathways, was employed for further functional interpretation [62,63]. Subsequently, KEGG pathway enrichment analysis was performed on the top ten hub genes using the DAVID database, applying a false discovery rate (FDR) threshold of <0.05.

We utilized the General Linear Model (GLM) [64] to investigate antiparallel gene expression, identifying genes with significant negative correlations across different days relative to the baseline. This analysis enabled the identification of genes exhibiting opposing expression patterns, with marked differential expression observed between distinct time points in the *Ssk12*-treated groups. DEGs were initially compared between day 4, corresponding to peak *Ssk12* colonization, and day 10, reflecting its decline. Genes with contrasting expression patterns between these conditions were extracted to compile a set of antiparallel DEGs identified through the GLM approach.

A non-parametric permutational multivariate analysis of variance (PERMANOVA) was conducted to assess overall differences in gene expression between day 4 and day 10 groups. Pearson’s correlation-based distance matrices were constructed from log counts per million (log_2_CPM) values for each gene using the *amap* package (v0.8-19) in R (http://CRAN.R-project.org/package=amap, accessed on 30 June 2025), serving as the basis for the analysis. Subsequently, Pearson’s product moment (PPM) correlation coefficients were calculated to evaluate the correlation of log_2_FC between the two groups. The absence of significant correlation or the presence of a negative correlation indicates nonparallelism in gene expression trends. Specifically, significant negative correlations highlighted antiparallel expression patterns, with certain genes exhibiting upregulation or downregulation corresponding to the peak and decline of *Ssk12* colonization.

### 4.9. Statistical Analysis

Data are presented as mean ± standard deviation (SD). Statistical differences between groups were analyzed using *GraphPad Prism* version 10.4.1 (GraphPad Software, San Diego, CA, USA). The Mann–Whitney U test was employed following an assessment of data normality, which was evaluated using quantile–quantile (Q–Q) plots within *Prism*. Due to the non-normal distribution of the data, non-parametric testing was applied. A two-tailed *p*-value < 0.05 was considered statistically significant.

To identify taxonomic differences associated with probiotic intervention, differential abundance analysis of 16S rRNA sequencing data was conducted using the *DESeq2* package in R (version 4.2). *DESeq2* was executed with default parameters, and multiple testing correction was applied using the Benjamini–Hochberg procedure to control the false discovery rate (FDR), implemented through the *MicrobiomeAnalystR* framework. Taxa exhibiting significant changes in abundance were further evaluated for biological relevance using the *LEfSe* method. *LEfSe* analysis was performed with default settings, applying a logarithmic LDA score threshold of 2.0 to identify discriminative features. A significance threshold of *p* < 0.05 was applied throughout to determine statistically meaningful differences.

## 5. Conclusions

This study presents the first integrated analysis of both compositional and functional alterations in the oral biofilm following probiotic colonization. A single-dose administration of the probiotic *Streptococcus salivarius* K12 resulted in transient colonization lasting up to 7 days, during which significant shifts were observed in the oral microbial community, including changes in both taxonomic composition and metabolic activity. These findings provide a foundational framework for the future optimization of probiotic delivery strategies aimed at achieving sustained colonization and enhanced therapeutic efficacy within the oral cavity. Furthermore, this study offers the first evidence supporting the use of three-dimensional melt-electrowritten (MEW) poly(ε-caprolactone) (*mPCL*) scaffolds as a physiologically relevant in vitro platform, capable of supporting the growth of saliva-derived polymicrobial biofilms with structural and functional features closely resembling those of native oral biofilms.

## Data Availability

The meta-transcriptomic sequencing datasets generated and analyzed during the present study are not publicly available at this time, as they form part of a larger research project from which additional publications are planned. To preserve the integrity of ongoing analyses and future data interpretations, public deposition has been deferred. However, the datasets are available from the corresponding author upon reasonable request.

## Supplementary Materials

The supporting information can be downloaded at: https://www.mdpi.com/article/10.3390/ijms26136403/s1. References [24,31,50,65,66,67] are cited in the supplementary materials.
