# Supplementary material for: Short-Term Probiotic Colonization Alters Molecular Dynamics of 3D Oral Biofilms
Source: Int J Mol Sci. 2025 Jul 3;26(13):6403. doi: 10.3390/ijms26136403 (PMC12249812; doi:10.3390/ijms26136403)
Supplement: Supplementary file 1 [file ijms-26-06403-s001.zip › ijms-3714952-supplementary.pdf]

Short-Term Probiotic Colonization Alters Molecular Dynamics of 3D Oral Biofilms

Supplementary Section 1 (S1)

Section.1.1: Manufacture of melt electrowriting MEW PCL scaffolds

Melt elctrowriting (MEW) was used to fabricate 3D fibrous scaffolds from medical-grade PCL (Corbion Inc., Australia, PURASORB PC 08, Item# 1850006). In brief, the syringe was preheated to a temperature of 85 ◦C for one hour before initiating the writing process to ensure a uniform mixture. Air pressure of 0.15 MPa was utilized to propel the polymer melt through the nozzle of the syringe. A voltage of 10 kV was applied using a DX250R apparatus from EMCO in Hallein, Austria, between the movable aluminium collector plate and the tip of the needle, with 8.0 mm between them. The MEW head, comprising the heater, syringe, and nozzle, remained stationary while straight fibers were deposited onto a moving collector plate at a speed of 1060 mm/min. The study involved the creation of square round-shaped scaffolds with 250 µm pore size and a thickness of 0.8 mm through melt electrowriting. Subsequently, the scaffolds were punched using a 5 mm punch to ensure identical dimensions in height and diameter for both MEW scaffolds. Custom translation patterns were generated using G-code for writing, and Mach3 motion control software from Artsoft in the USA was employed to manage the parameters.

Section.1.2: Rationale for selecting scaffolds with 250 µm pore size and 4-day- old salivary biofilm cultured on MEW PCL scaffold for the proposed study

Based on our previous study [1], the cross-sectional scanning electron microscopy (SEM) examination of saliva cultured on MEW250 scaffolds on day 10 revealed the presence of pores and channels that are typical features of bacterial biofilms. Notably, exopolymeric bridges, which indicate interactions among bacterial aggregates within the matrix, were prominently observed on the MEW250 scaffolds. This finding underscores the appropriateness of MEW250's pore size for examining the long-term effects of probiotic colonization within the 3D biofilm model proposed in the current study.

Moreover, 16S rRNA sequencing of the 4-day-old biofilms revealed that a significant proportion of bacterial phyla and genera reatained around 60% of the core microbiome at both the phyla and genus levels within the 3D MEW PCL substrates [1]. This preservation suggests the establishment of a core microbiome when contrasted with the original saliva samples prior to culturing, highlighting the rapid growth and capability to develop mature biofilms with polymicrobial features.
Consequently, day four was selected as the baseline time point for evaluating the impact of probiotic bacteria on the colonization and growth of salivary biofilms. The experimental time points were defined as days 4, 7, and 10, measured after the baseline.

Section.1.3: Sample size justification

Although our study employs a small sample size of four healthy participants, this approach is justified given the exploratory nature of the research and the relatively low biological variability expected in healthy individuals. Additionally, the homogeneity of our samples derived from healthy participant minimizes variability, allowing for meaningful insights into the effects of probiotic intervention under study. To further enhance the reliability of our findings, we incorporated rigorous quality control measures, including [technical replicates, increased sequencing depth (>10 million reads/sample), rigourous statistical methods], which have been shown to compensate for smaller sample sizes in metagenomic studies [2, 3].

Section.1.4: Salivary Biofilm culture and Ssk12 inoculation

Ssk12 strain was incubated at 37°C in an orbital shaker set at 150 rpm for 18 hours. 1ml of *S. salivarius* (1 x 10^7 bacteria/ml) from an overnight culture was introduced into 10 ml of BHI broth, and the bacterial suspension was left to incubate for 24 hours under aerobic conditions. After incubation, the K12 suspension underwent centrifugation at 5000g for 10 minutes, and the resulting supernatant was transferred to a new 15 ml conical tube. Following this step, the supernatant was filtered using a polyvinylidene fluoride filter (Millipore, Billerica, MA, USA). The filtered supernatant, referred to as spent culture medium (SCM), was utilized for subsequent salivary biofilm culture assays.

The MEW PCL scaffolds were sterilized through immersion in 70% ethanol for a duration of 15 minutes, followed by ultraviolet sterilization for 20 minutes. Saliva samples from four healthy participants were individually collected to serve as inoculum for biofilm culture, utilizing a combination of defibrinated sheep's blood (ThermoFisher Scientific) and heart infusion (HI) media at a ratio of 1:1:8. A volume of 300 µl from these mixtures was inoculated into 48 wells, both with and without MEW mPCL scaffolds, and subsequently incubated for 10 days within an anaerobic chamber (Whitely A20 anaerobic chamber) at 37 °C on a large shaker rotating at 80 rpm. Approximately 15 mL of the conditioned media from the MEW mPCL scaffolds were then collected for the isolation of DNA and RNA.

Section.1.5: Characterization of salivary biofilm biomass

The XTT assay measured the metabolic activity of the biofilm. After two PBS washes, 300 µl of activated XTT solution (Sigma-Aldrich) was added to each well, incubated in the dark at 37°C for four hours, and absorbance was measured at 492 nm using a TECAN Infinite M200PRO plate reader.

The crystal violet assay quantified total biofilm formation, both in the presence and absence of biofilm. Biofilms cultured on MEW mPCL and TCP surfaces were stained with 0.1% crystal violet (Sigma-Aldrich, Australia) for 15 minutes, followed by a 10% (v/v) acetic acid treatment for 15 minutes to solubilize the bound dye. Absorbance was then measured at 570 nm on the TECAN Infinite M200PRO.

To assess bacterial viability within saliva-based biofilms, a LIVE/DEAD BacLight™ bacterial viability kit (Life Technologies, Australia) was used for biofilm staining. A solution containing 3 µl SYTO™ 9 and 3 µl propidium iodide (PI) was added to 1 ml of PBS per well, followed by a 30-minute incubation. Biofilm visualization was conducted using a confocal microscope (Leica TCS SP5). The percentage of green fluorescence (live bacteria) and red fluorescence (dead bacteria) was analyzed in each field using ImageJ software. The live/dead ratio of the biofilm was calculated using the following equation.

$$Live\ or\ Dead\% = \frac{\%Green\ (Live)\ or\ \%Red\ (dead)\ area}{\%Green\ (Live) +\ \%Red\ (dead)\ area} \times 100\%$$

Section.1.6: Validation of colonization Ssk12 in salivary biofilm biomass using real-time quantitative PCR (RT-PCR) and smFISH (single molecule FISH)

The successful identification of Ssk12 was confirmed only when all three targets (*sboA*, *sboG*, and *sboK*) were amplified in the sample as previously described [4]. The amplification was carried out using extracted 5 µl of total genomic DNA template (1 ng/µl) in accordance with previously established protocol 5. The assay involved a duplex TaqMan™ setup containing forward and reverse primers for *sboA* and *sboG* (600 nM), along with probes for *sboA* and *sboG* (200 nM). Additionally, a singleplex TaqMan™ configuration included forward and reverse

primers for *sboK* (600 nM) and a *sboK* probe (200 nM). The TaqMan qPCR assays were performed on a QuantStudio 6 Flex Real-Time System (Applied Biosystems) with the following thermal cycling parameters: an initial denaturation at 95 °C for 10 minutes, followed by 40 cycles of 95°C for 15 seconds and 60°C for 1 minute.

Each experiment was conducted in triplicate, with strain K12 genomic DNA and K12 colony (500x diluted) serving as positive controls, and sterile deionized water as the negative (no template) control, as optimized in previous study 5.

While the study was conducted through to the 14th day (data not shown); however, as no Ssk12 colonization was observed beyond the 10th day, this was designated as the endpoint for the further downstream analysis. We further investigated colonization potential of naturally occurring *Ssk12* would dissociate within the 3D MEW mPCL scaffold over the specified time. Colonization of naturally occurring *Ssk12* was not detected beyond the 7th day (data not shown).

In *smFISH* hybridization [5], Salivary biofilm-coated MEW mPCL scaffolds were 4% formaldehyde in 1x PBS for 10 min at room temperature. Remove formaldehyde and then wash once with 135mM Glycine in 1x PBS to quench residual formaldehyde for 10 min. Samples were incubated overnight in a humidified chamber at 37 °C in HuluHyb solution (2xSSC, 2M Urea, 10% dextran sulfate, 5x Denhardt's solution) containing 1 ng/µL HuluFISH probes. Samples were then incubated at 37°C for 30 min, with HuluWash buffer, followed by a further two washes to remove nonspecific binding of the probe. Substrates were counterstained with DAPI (Invitrogen).

Section.1.7: Microbial profile using 16srRNA sequencing mRNA sequencing

In DNA extraction, briefly, PCL Substrates with salivary biofilms were removed from the tubes, which were then centrifuged at 10,000 g for 10 minutes at 4 ºC. Subsequently, the supernatant was carefully decanted and discarded. To lyse the cells, S1 (lysis buffer) and S2 (lysis enhancer) were utilized, while beads (0·070–0·125 mm; provided in the kit) facilitated mechanical lysis. These beads were transferred to bead tubes, followed by an incubation at 65 ºC. The samples were then centrifuged at 4000 g for 2 minutes at 4 ºC to pellet the DNA. The pelleted DNA was mixed with binding buffer, washed twice with 75% ethanol, and ultimately eluted with TE buffer. The quality of the extracted genomic DNA (gDNA) was assessed based on purity, indicated by the A260 nm/A280 nm ratio, using the Nanodrop (Thermo Scientific™).

NanoDrop™ One Microvolume UV-Vis Spectrophotometer. Additionally, the quantity of DNA concentration in the extracts was determined using the Qubit 2·0 Fluorimeter (ds DNA high-sensitivity assays kit; Invitrogen). The gDNA with an A260/280 ratio of 1.8 was further processed for both RT-PCR quantification and 16S rRNA next- generation sequencing.

In RNA extraction, the substrates were first subjected to additional purification steps involving lysozyme treatment and bead beating. Previous research [6] has shown that this pre-treatment, in conjunction with a column-based kit, yielded the most favorable results by exhibiting the highest relative abundance of bacteria and functional genes in multi- species biofilm samples.

To initiate the process, a 60-µl volume of lysozyme solution (5 mg/ml) from Sigma (L6876-1G) was introduced to the substrate, which had been rinsed with PBS liquid biofilm sample. Additionally, 30 µl of NaCl-EDTA solution (0.3 M, 0.02 M) was added as the activating buffer. The samples were then subjected to two cycles of disruption, involving 40 seconds of vortexing followed by 20 seconds of rest, using glass beads measuring 0.150 to 0.212 mm (Sigma, G1145-100G). Subsequently, RNA extraction was performed using standard RNAzol methodology. Specifically, 1 ml of RNAzol®RT was combined with 0.4 ml of the liquid sample, and the resulting

RNA-containing supernatant was transferred to a new tube. The RNA was precipitated by mixing 1 ml of the supernatant with an equal amount of isopropanol, followed by centrifugation at 12,000 g for 10 minutes. After washing the RNA pellet with 75% ethanol (v/v) and centrifuging at 4,000 - 8,000 g for 1 - 3 minutes, the RNA pellet was dissolved in RNAse-free water without drying. Quality control measures were applied to the total RNA through NanoDrop analysis (Thermo Fisher, United States) for assessing RNA purity (OD260/OD280; OD260/OD230), Qubit analysis (Thermo Fisher, United States) for determining RNA yield, and Agilent 2100 Bioanalyzer analysis (Agilent Technologies) for verifying RNA integrity. RNA extracted from each sample was pooled in triplicates to ensure the highest quality of RNA, with a RIN number ranging from 6.8 to 8 and an A260/280 ratio between 2 and 2.2, before being sent for library construction and subsequent meta- transcriptomic sequencing and analysis.

In 16s rRNA sequencing, initial sequencing runs underwent quality control assessments using Illumina's Sequencing Evaluation Viewer version 2.4.7. Double-end sequencing of both positive and negative reads was performed, with the initial read of each pair being combined to exclude results that contained N sequences. Following the quality filtering process, sequences exceeding 200 base pairs in length were retained. Chimeric sequences were eliminated, and the resulting sequences were utilized for operational taxonomic unit (OTU) clustering. VSEARCH clustering version 1.9.6 was applied to group sequences with a similarity threshold of 97%. The representative sequences of each OTU were subsequently compared against the 16S rRNA reference database Silva version 6.

In mRNA sequencing, RNA-Seq libraries were prepared using the Illumina stranded total RNA prep ligation with an IDT for Illumina RNA UD Indexes (illumina, 20040554) according to the standard manufacturer's protocol (illumina, Document # 1000000124514 v03, June 2022) described briefly as follows. 80 ng of total RNA was depleted of abundant rRNA and then fragmented in a heat fragmentation step. cDNA was synthesized from the fragmented RNA using random primers. The first strand cDNA was converted into dsDNA in the presence of dUTP to prevent subsequent amplification of the second strand and thus maintaining the strand orientation of the original RNA. The 3' ends of the cDNA were adenylated and pre-index anchors were ligated. The libraries were then amplified with 14 cycles of PCR incorporating unique indexes for each sample to produce libraries ready for sequencing. The libraries were quantified on the Perkin Elmer LabChip GX Touch with the DNA High Sensitivity Reagent kit (Perkin Elmer, CLS760672). Libraries were pooled in equimolar ratios. Sequencing was performed using the Illumina NextSeq500 (NextSeq control software v4.0.0 / Real Time Analysis v2.11.3). The library pool was diluted and denatured according to the standard NextSeq protocol (Document # 15048776 v16) and sequenced to generate single-end 82 bp reads using a 150 cycle NextSeq500/550 High Output reagent Kit v2.5 (illumina, 20024907) (12-16 million reads/ sample).

When megaHit is assembled, multiple parameters of K-mer = 21 ~ 141 are selected for assembly, and then the optimal result is selected to obtain the final assembly result. Scaffold generated by mixed assembly, retains sequences longer than 500bp, and performs statistical analysis and subsequent gene prediction. Trimmed RNA-seq reads were mapped against the reference genome by HISAT2 7. COG IDs offer a systematic and efficient framework for classifying bacterial gene clusters based on their functional roles. By grouping genes into orthologous clusters, researchers can infer the functional significance of genes in newly sequenced bacterial genomes. This approach enables the identification and categorization of bacterial genes according to their predicted functions and associated protein domains, thereby providing valuable insights into the biological processes they may influence.

Supplementary Section 2 (S2)

Table S1. A) Data are displayed as mean ± standard deviations. BOP:bleeding on probing, and PI: plaque index. B) Visual timeline of Experimental setup.

	Participants (n=4)	
**Gender**	Male	2 (50%)
	Female	2 (50%)
**Age**	38.5 ± 1.29 (37, 40)	
**BOP**	1.8% ± 0.54 (1%, 2.2%)	
**PI**	0.075% ± 0.07 (0%, 0.18%)	
**Ethnicity**	Asian (100%)	

Table S2. Differentially abundant genera compared to the baseline group were identified using DESeq2 and LEfSe analysis based on 16S sequencing data. A) DESeq2 analysis was performed with default settings, and q-values were calculated using the Benjamini-Hochberg procedure in R (version 4.2). Genera highlighted in red indicates significant results with q < 0.05. B) Differentially abundant microbial biomarkers were identified using the Linear Discriminant Analysis Effect Size (LEfSe) method with default parameters, setting the LDA log score threshold at 2. A p-value of less than 0.05 was considered statistically significant, accounting for both significance and biological relevance.

A)

Group	Feature	log2FC	lfcSE	Pvalues	FDR
Day 4	Actinomyces	5.2192	2.09	0.012518	0.013769
Day 4	Lactobacillus	-0.81034	1.014	0.03242	0.0363142
Day 4	Staphylococcus	2.0298	1.9662	0.3019	0.4663142
Day 4	Bifidobacterium	2.2722	2.4325	0.35025	0.463142
Day 4	Streptococcus	-0.46478	0.77546	0.4254893	0.4663142
Day 4	Alloscardovia	2.246	3.3312	0.4250016	0.4663142
Day 4	Veillonella	-0.80506	1.1693	0.429115	0.463142
Day 7	Actinomyces	1.0219	1.8896	0.458865	0.468887
Day 7	Lactobacillus	-0.89074	1.0145	0.37996	0.468887
Day 7	Staphylococcus	5.1299	2.1527	0.17174	0.18891
Day 7	Bifidobacterium	1.9521	2.4325	0.42226	0.468887
Day 7	Streptococcus	-0.5322	0.77959	0.49482	0.68887
Day 7	Alloscardovia	1.9259	3.3312	0.56317	0.68887
Day 7	Veillonella	-1.3034	1.169	0.26488	0.68887
Day 10	Actinomyces	6.3819	2.1302	0.0027363	0.030099
Day 10	Lactobacillus	0.52321	1.0206	0.046082	0.0369145
Day 10	Staphylococcus	-2.9545	1.9404	0.012785	0.032252
Day 10	Bifidobacterium	-4.9349	2.2142	0.025831	0.014207
Day 10	Streptococcus	0.68204	0.81285	0.40143	0.63081
Day 10	Alloscardovia	-3.1644	3.1856	0.32054	0.63081
Day 10	Veillonella	1.6891	1.1839	0.15364	0.42252

B)

Feature	Group	LDA score	FDR
**Bifidobacterium**	Day 10	4.75	0.0490906
**Staphylococcus**	Day 10	5.45	0.0197497
**Alloscardovia**	Day 10	4.24	0.012708
**Actinomyces**	Day 4	4.63	0.022392
**Veillonella**	Day 4	5.22	0.023425
**Lactobacillus**	Day 4	5.17	0.023425
**Streptococcus**	Day 4	4.47	0.039504

Figure S1. Characterization for biofilm cultured on 3D mPCL scaffold. A) Confocal imaging of the 3D PCL biofilm reveals a comprehensive 3D Z-stack and sectional views, indicating that the thickness of the salivary biofilms reaches approximately ~100-300 μm at all observed time points in the absence of *Ssk12* treatment. B) A bar plot illustrates the changes in salivary biofilm thickness across the 3D mPCL scaffolds (MEW 250) with *Ssk12* treatment, measured at each intended point. No significant differences in biofilm thickness were observed with *Ssk12* treatment compared to baseline.C) The boxplot presents the relative abundance of Streptococcus salivarius across baseline (no probiotic) and post- inoculation time points (days 4, 7, and 10). A significant temporal variation was detected (Kruskal–Wallis, p = 0.033), with a pronounced increase at day 4, indicative of peak S. salivarius K12 (Ssk12) colonization. Data points represent individual samples, while boxplot elements display the interquartile range, median, and 1.5× IQR whiskers.

A)

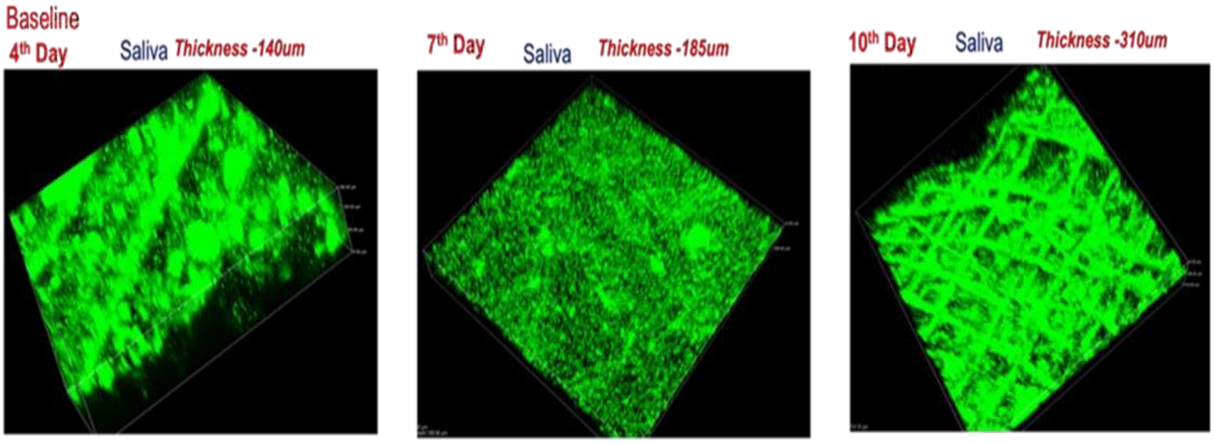

B)

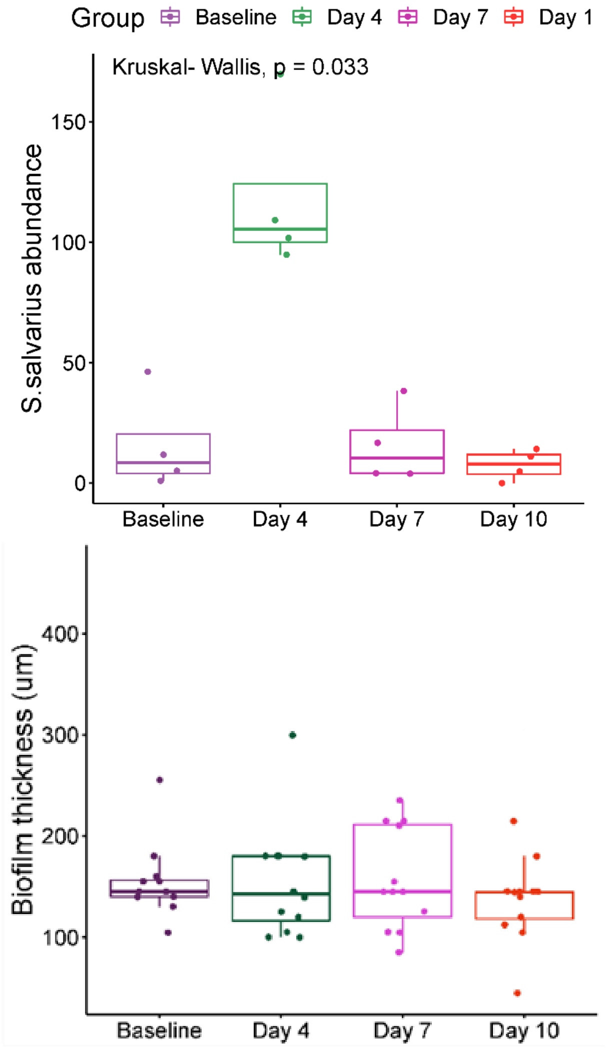

Figure S2. Multivariate sparse partial least-squares discriminant analysis based on 16s sequencing. (sPLS-DA) of read counts transformed with log-cumulative-sum scaling at the level of genus variants using 16S rRNA gene amplicon sequencing. A) Genera contributing to separation with component-1 of sPLS-DA (red dotted squares indicating most discriminative genera). B) Rank of the importance of loading coefficients at the genus level. (most discriminative genera are shown in red dotted squares), bottom to top; the colour of the bar indicates the group where the sequence variant has the highest median abundance based on component 1. The heat map of read counts is transformed using log-cumulative-sum scaling to identify discriminatory sequence variants along with component −1 of sPLS-DA.

A)

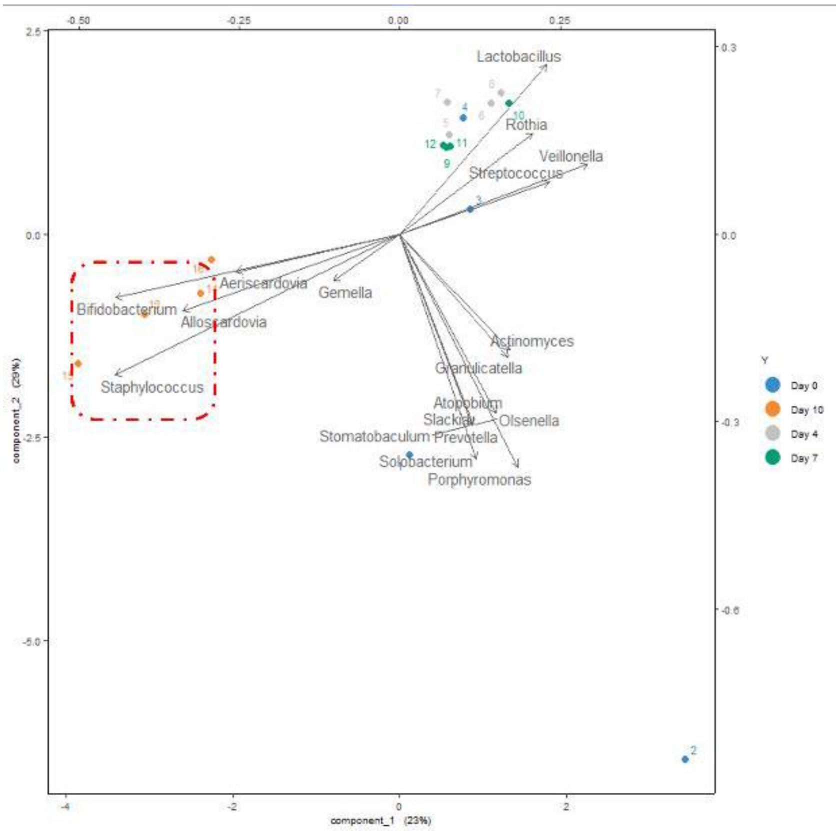

B)

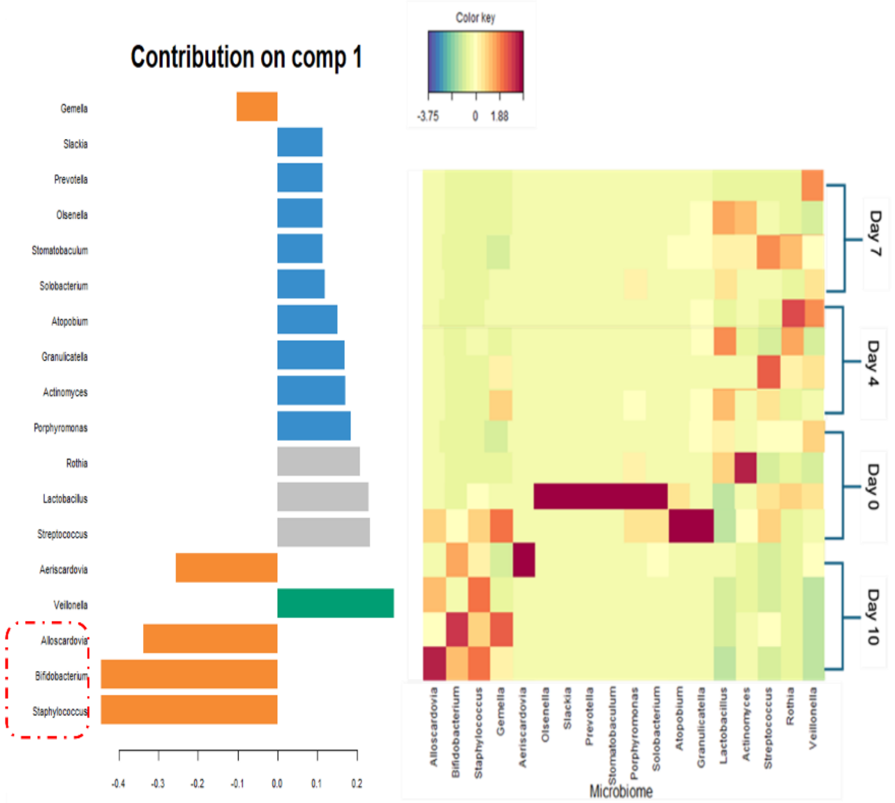

Figure S3. **Bacterial composition and gene distribution.** A) The dendrogram illustrates the clustering of samples based on similarity in their microbial composition over the Baseline, Day 4, Day 7, and Day 10. The vertical axis ("Height") reflects the level of dissimilarity or distance between clusters, with higher branches indicating greater differences between groups. Day 10 and most of Day 4 samples tend to form separate clusters, showing differences in community composition before stabilizing or evolving further. Further, this indicates that microbial communities are not static and may adapt or respond to the probiotic over the study period. B) The UpSet plot provided illustrates the intersection sizes of DEGs (Differentially expressed genes) between baseline and subsequent days in the study (Day 4, Day 7, and Day 10), with the y-axis representing the size of these intersections. This plot suggests that a large core set of DEGs (952) unique to Baseline vs. Day 10, second largest DEGs (515) unique to Baseline vs. Day 4 indicate specific fluctuations in these genes that may reflect adaptive responses to probiotic treatment, while fewer DEGs persists across all days suggest stabilization in functional diversity over time.

A)

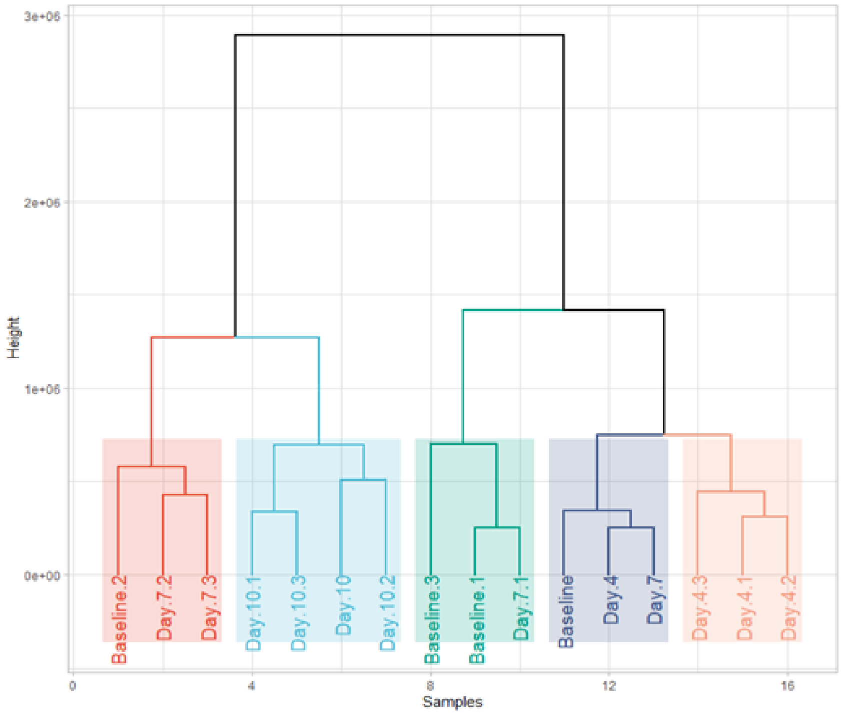

B)

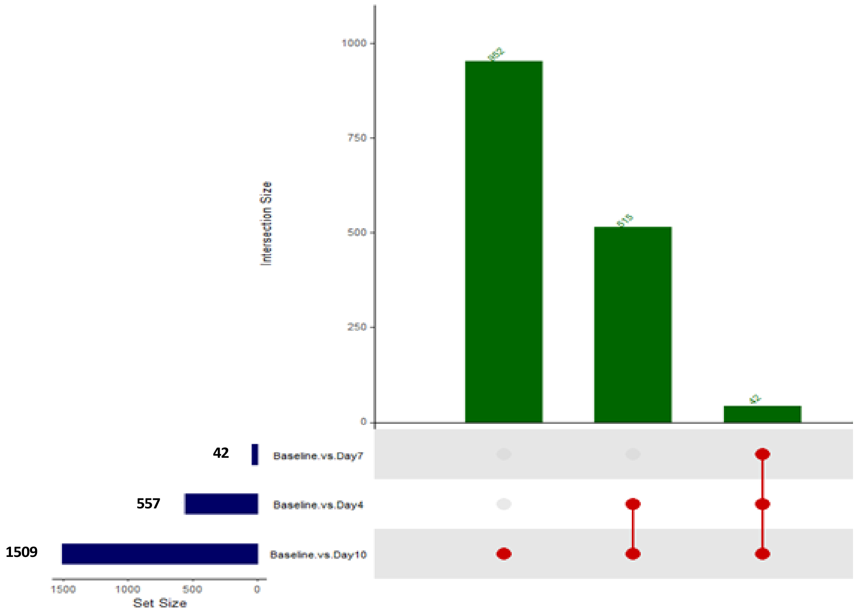

Figure S4. Sankey plot showing energy-intensive metabolic processes connected with the genes along with their associated −log10P values. A) Identified at day 4. B) Identified at day 10. Each gene and pathway are represented as a node, with genes typically on one side of the plot and pathways on the other. Higher −log10P (higher intensity of color) values correspond to smaller *P*-values, meaning stronger statistical significance. Gene counts to particular pathway represented by size of the circle. Genes with connections to numerous pathways can indicate pleiotropic effects, meaning they impact multiple pathways and thus may play a more central role in biological functions.

A)

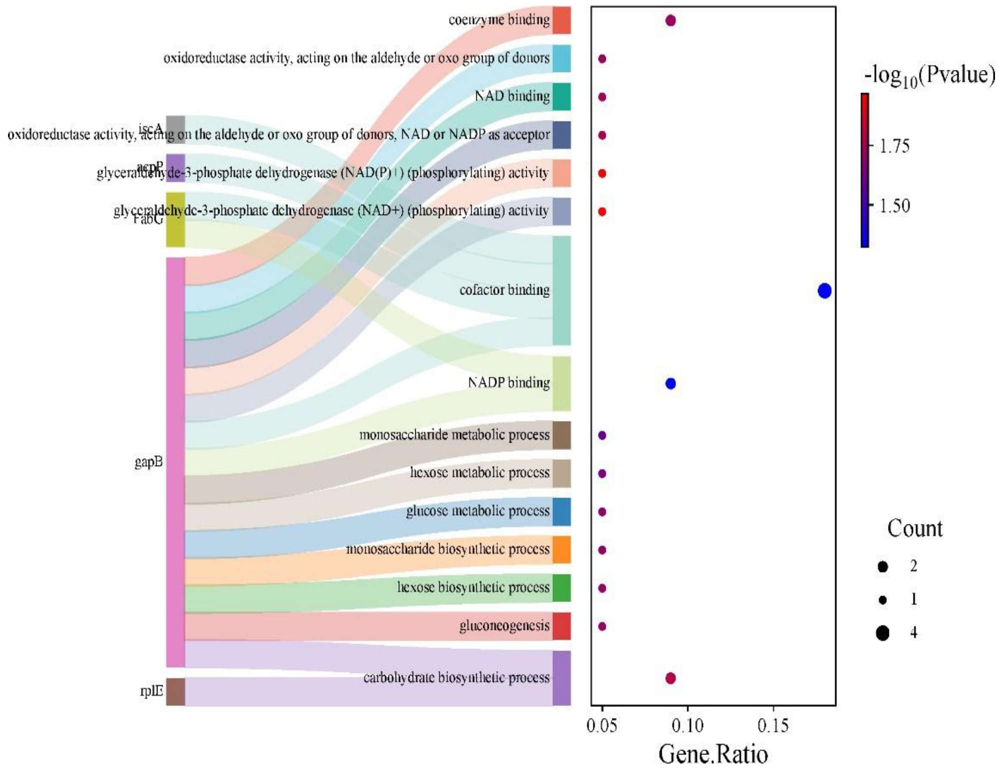

B)

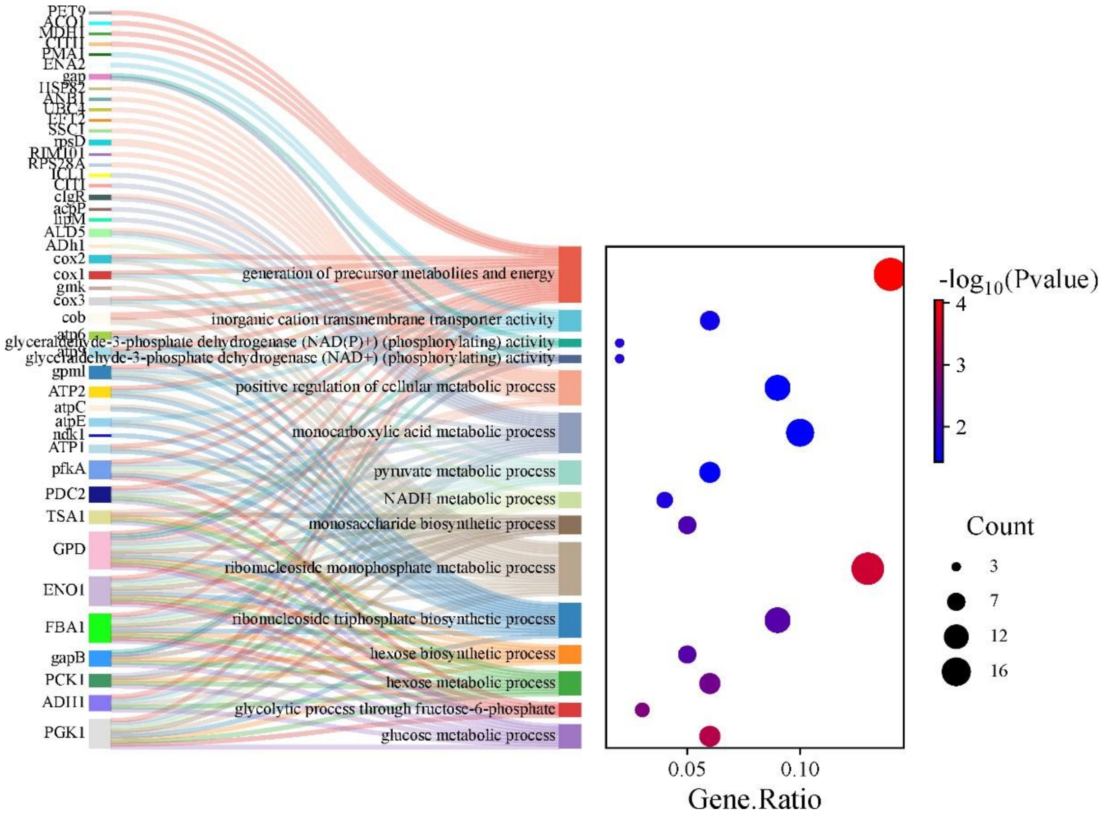

Figure S5. **The protein-protein interaction (PPI) networks of differentially expressed genes (DEGs) and key modules on days 4 and 10 reveal essential relationships between proteins.** In these PPI networks, nodes represent proteins, and edges denote interactions, often weighted by confidence scores. The Molecular Complex Detection (MCODE) algorithm identifies clusters, or modules, within the PPI network based on connectivity and density, assigning a score to each module according to its clustering coefficient; higher scores indicate more densely interconnected protein groups analyzed using STRING database. Modules with highest scores reflect high intramodular connectivity and imply a coordinated regulatory or functional association among the genes within the cluster whereas modules with moderate scores indicate condition-specific gene activity or reflect underlying heterogeneity in biological responses across the system. A) On day 4, the MCODE plugin of Cytoscape identified 2 significant gene cluster modules based on unique 557 DEGs. The significant two key modules from the PPI network were selected: Module 1 contained 99 nodes and 3552 edges with a score of 72.49, Module 2 comprised 75 nodes and 1017 edges with a score of 27.486. D) On day 10, the MCODE plugin of Cytoscape identified 3 significant gene cluster modules based on 970DEGs. The significant 3 key modules from the PPI network were selected: Module 1 contained 142 nodes and 8391 edges with a score of 119.021. Module 2 comprised 61 nodes and 513 edges with a score of 27.486. comprised 64 nodes and 505 edges with a score of 16.032. Tables (B) and (E) illustrate these key modules, where highly connected proteins likely play vital roles in cellular functions or pathway regulation, often serving as key drivers in signaling or metabolic pathways. Tables (C) and (F) show the top 5 hub genes for each day's PPI network, which represent the most central or influential genes (nodes) in the network. These hub genes are frequently linked to critical pathways, disease mechanisms, or cellular structural maintenance. Notably, genes such as *budC*, *fabG*, *sigH*, *sigS*, and *tetR*, which are shared between days 4 and 10, appear to be pivotal in maintaining essential cellular functions and stabilizing network structure under probiotic modulation, highlighting pathways fundamental to cellular survival and adaptability.

A)

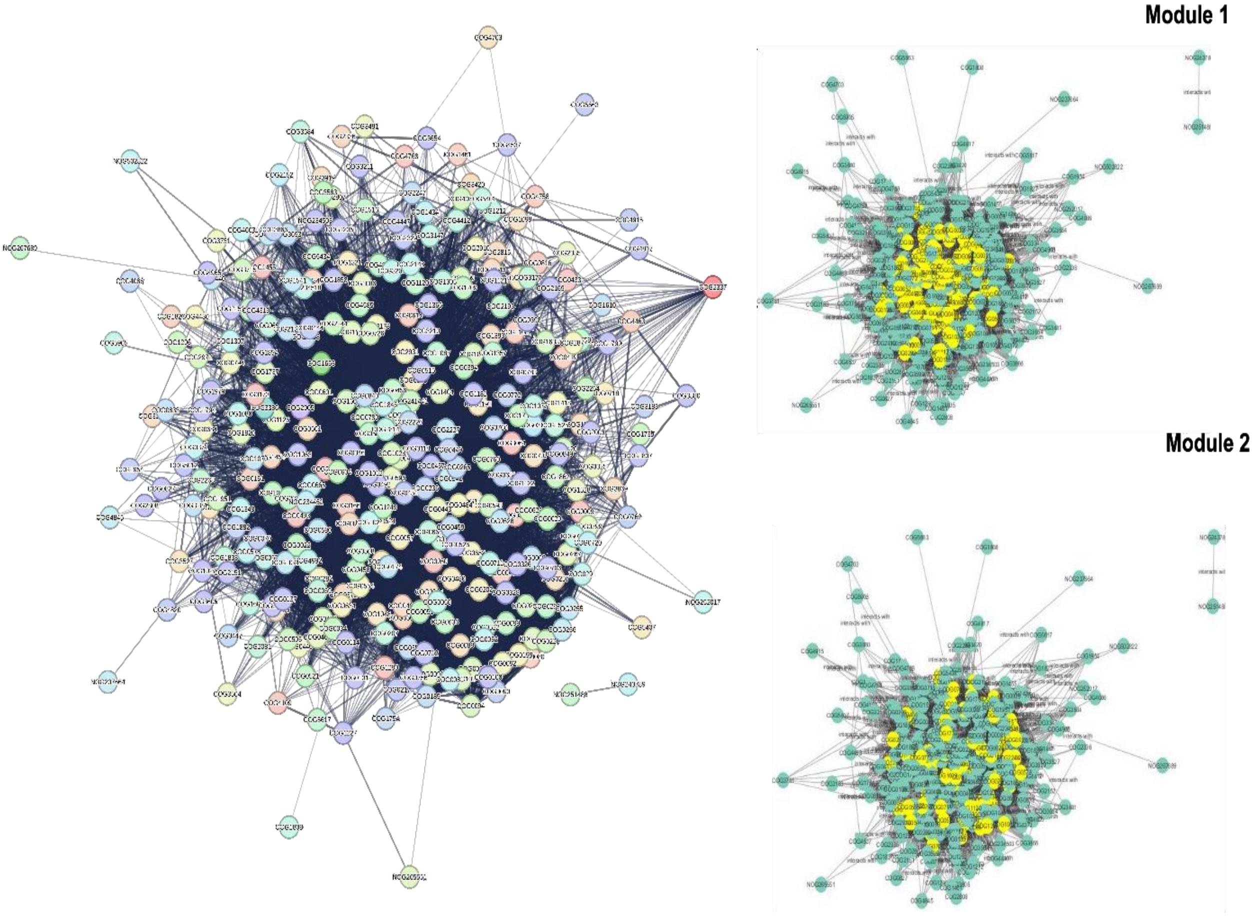

B)

Top COGID	Module	Identified Genes
COG1028	Module 1	fabG, budC
COG1309	Module 1	tetR
COG0583	Module 1	lysR, ywbl, ccpC, gltC
COG1595	Module 1	sigH, sigS
COG0642	Module 2	nsaS,graS,yclK
COG1012	Module 2	gabD
COG0457	Module 2	yrrB,gluP,STI1,SSN6,SSN6
COG1609	Module 2	araR,malR,ccpA,rbsR,purR2,scrR,rbsR

C)

Top Hub COGID	Degree	Identified Genes	Closeness	Betweenness	Stress	Clustering Coefficient
COG1028	266	fabG, budC	286.5	3944.815	53030	0.32058
COG0642	257	nsaS,graS,yclK	282	3169.886	48638	0.33275
COG1309	256	tetR	281.5	3191.183	50592	0.31048
COG2226	234	sigH	270.5	2024.006	38212	0.34881
COG1595	231	sigH, sigS	269	2136.276	37846	0.3447

D)

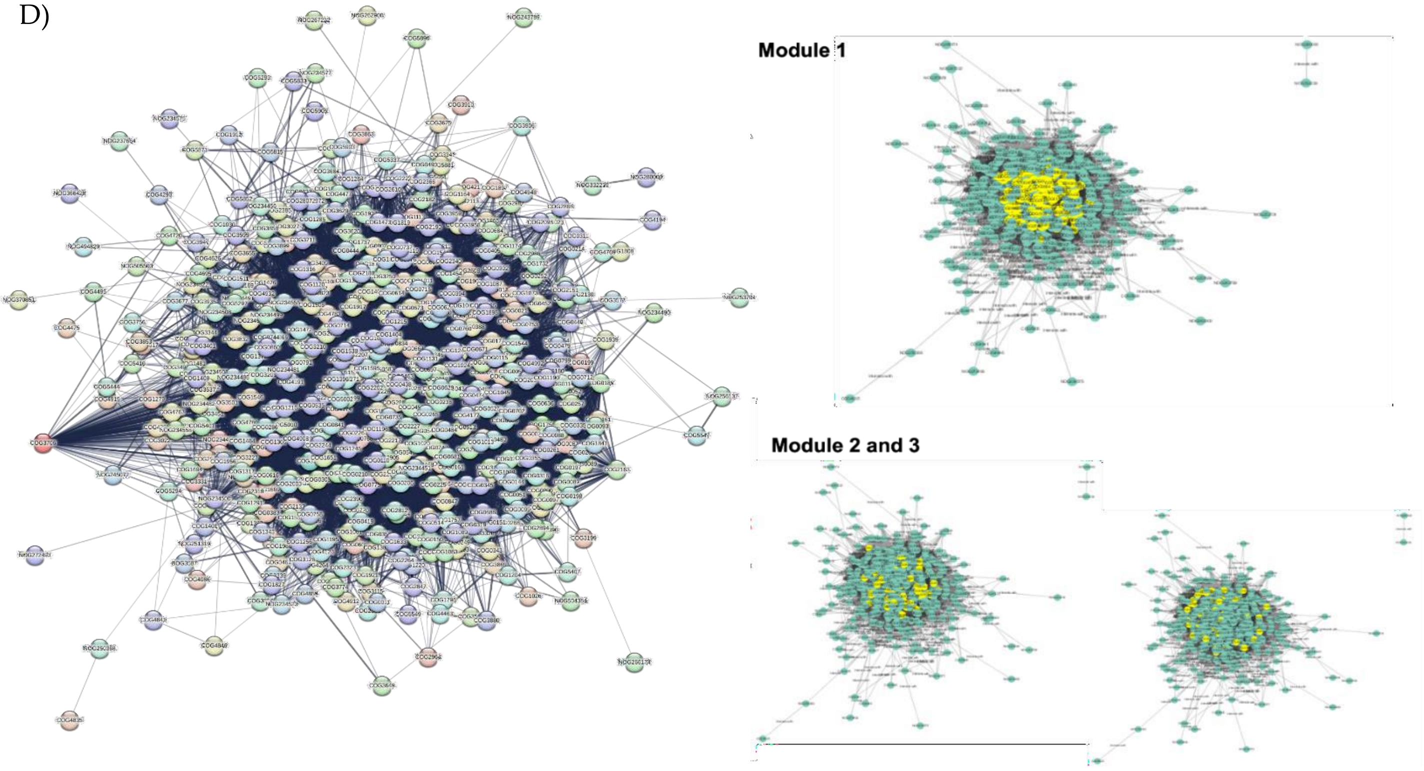

E)

Top COGID	Module	Identified Genes
COG0515	Module 1	prkC,pknk,pknB
COG0463	Module 1	ykoT, crtQ
COG0745	Module 1	rlrB, mleR2, ywbl,ccpC,gltC
COG0583	Module 1	rlrB,mleR2,ccpC,gltC
COG0770	Module 2	murE2,murF
COG1595	Module 2	sigH, sigS
COG0514	Module 2	recQ
COG1185	Module 2	pnp
COG1366	Module 3	rsbV
COG1086	Module 3	capE,capD
COG0628	Module 3	yubA,yueF,ydbl
COG0206	Module 3	ftsZ

F)

Hub COGID	Degree	Identified Genes	Closeness	Betweenness	Stress	Clustering Coefficient
COG0745	401	rlrB, mleR2, ywbl, ccpC, gltC	450.3333	9288.359	169046	0.2983
COG1028	381	fabG, budC	438.9167	5492.583	132670	0.32351
COG1309	377	tetR	437.25	5891.004	138268	0.31747
COG0583	363	rlrB,mleR2,ccpC,gltC	429.9167	4852.673	122056	0.33219
COG1595	363	sigH, sigS	430.5833	5772.95	133720	0.33081

Figure S6. KEGG pathway enrichment analysis was conducted on the top three gene modules identified from the PPI network using the MCODE algorithm based on differentially expressed gene (DEG) profiles. (A) At day 4, module 1 showed enrichment in 13 KEGG pathways, while module 2 was associated with 11 distinct pathways. (B) By day 10, module 1 exhibited enrichment in 8 KEGG pathways, whereas modules 2 and 3 were each linked to 11 KEGG pathways.

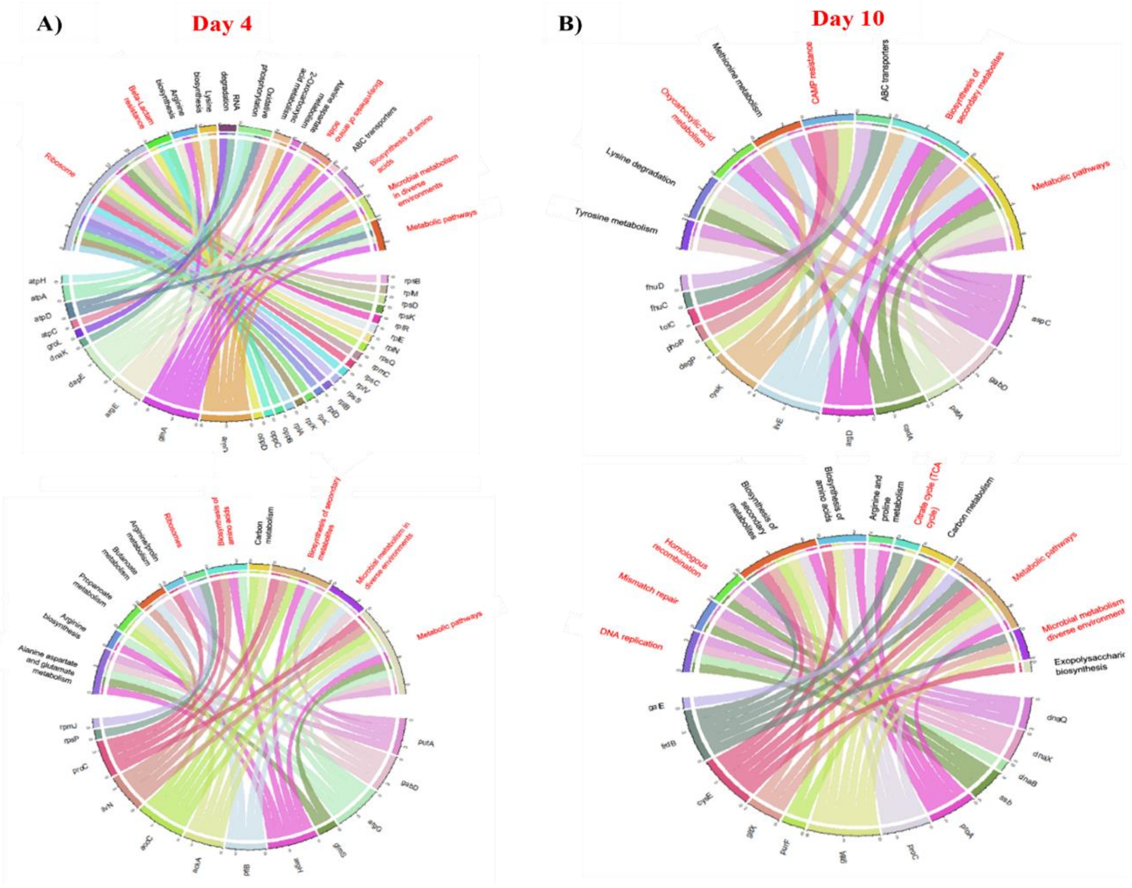

Figure S7. **Assessing the level of gene correlation gene between peak S. sK12 colonization and its subsequent decline.** (A) The Sankey plot visualizes dynamic shifts based on the most significant antiparallel genes and pathway activities between days 4 and 10 (refer to main Figure 3f). Genes associated with each pathway are listed on the right side, including rplE, pfkA, gapB, acpP, PGK1, ENO1, and ADH1. Each gene is linked to specific pathways, highlighting its role in particular cellular processes. Notably, genes such as PGK1, ENO1, and ADH1 are connected to multiple pathways, underscoring their central roles in energy-intensive metabolic functions across both time points.B) This heatmap presents gene correlation based on overall gene expression between days 4 and 10 using Spearman's correlation coefficients. The color gradient from purple to red represents the correlation strength, with purple (close to 0) indicating no correlation, and deep red (close to 1) indicating a strong positive correlation. Negative correlations, although present, are relatively weak in this dataset and are shaded in lighter purples. The heatmap shows that samples from day 4 are highly correlated with each other, as are samples from day 10, indicating homogeneity within each group. In contrast, weak inter- group correlations suggest distinct gene expression differences between these two points.

A)

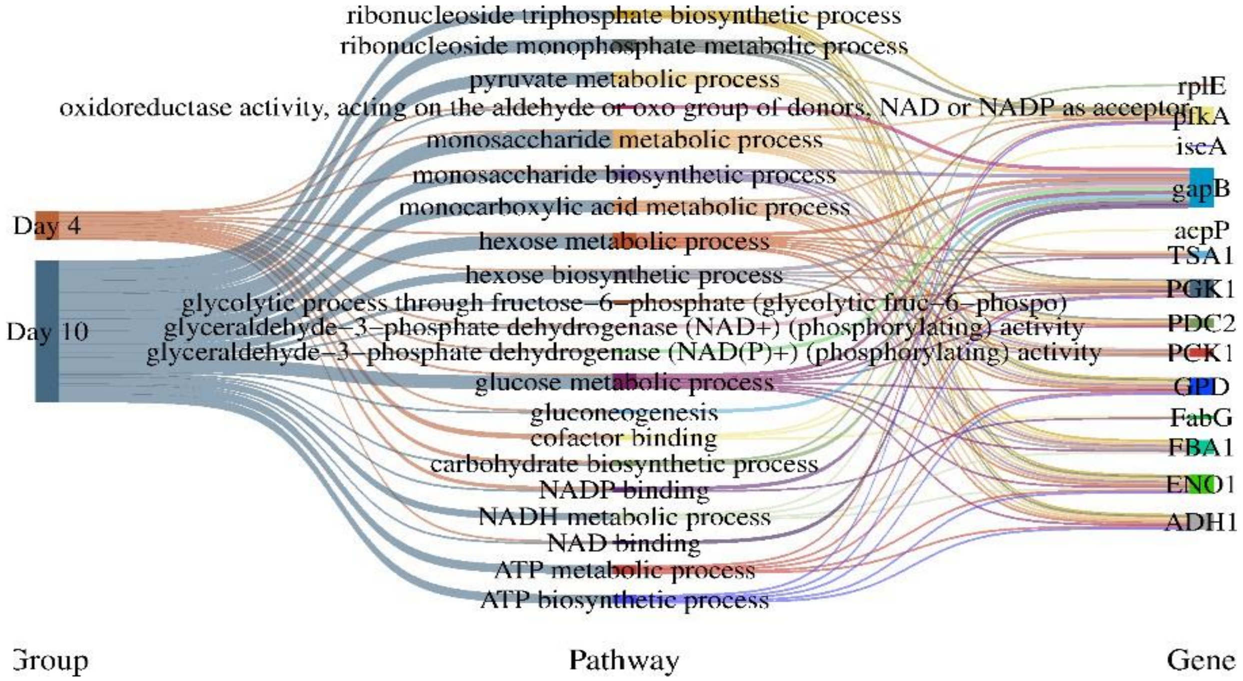

B)

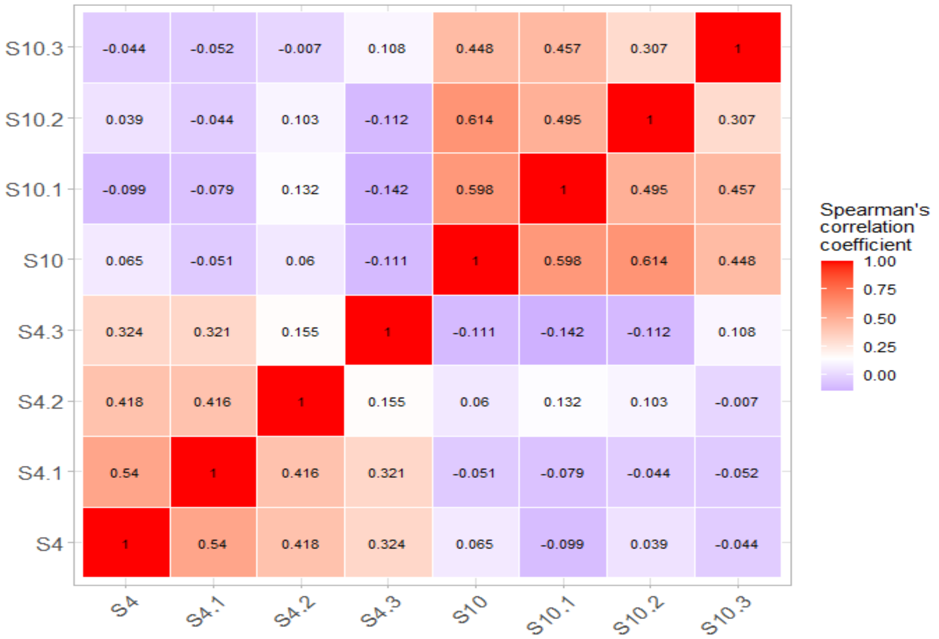

References

1. Ramachandra, S. S., Abdal-Hay, A., & others. (2023). Fabrication and characterization of a 3D polymicrobial microcosm biofilm model using melt electrowritten scaffolds. Biomaterials Advances, 145, 213251. https://doi.org/10.1016/j.bioadv.2022.213251.
2. Schurch, N. J., Schofield, P., Gierliński, M., Cole, C., Sherstnev, A., Singh, V., ... & Barton, G. J. (2016). How many biological replicates are needed in an RNA-seq experiment and which differential expression tool should you use? RNA, 22(6), 839–851. https://doi.org/10.1261/rna.053959.115.
3. Liu, Y., Zhou, J., & White, K. P. (2014). RNA-seq differential expression studies: More sequence or more replication? Bioinformatics, 30(3), 301–304. https://doi.org/10.1093/bioinformatics/btt688.
4. Horz, H. P., Meinelt, A., Houben, B., & Conrads, G. (2007). Distribution and persistence of probiotic Streptococcus salivarius K12 in the human oral cavity as determined by real-time quantitative polymerase chain reaction. Oral Microbiology and Immunology, 22(2), 126–130. https://doi.org/10.1111/j.1399-302X.2007.00334.x.
5. Han, K., Liu, S., & Cheng, Y.-S. (2021). HuluFISH non-denaturing in situ detection of genomic DNA opened by CRISPR-Cas9 Nickase and Exonuclease. bioRxiv. https://doi.org/10.1101/2021.08.10.455844
6. Yao, Y., Rao, S., & Habimana, O. (2021). Active microbiome structure and functional analyses of freshwater benthic biofilm samples influenced by RNA extraction methods. Frontiers in Microbiology, 12, 588025. https://doi.org/10.3389/fmicb.2021.588025.
